# Engineered TIGIT‐Blockade Membrane Vesicles Synergize with Microwave Ablation to Mediate Liver Metastases Eradication

**DOI:** 10.1002/advs.202522918

**Published:** 2026-02-08

**Authors:** Shaoyue Li, Weichen Xu, Yuting Shen, Xuexia Shan, Jiawei Sun, Shisi Ding, Xiaodong Hou, Shaoning Zhang, Zhiyuan Niu, Taixia Wang, Xin Guan, Xiao Li, Weiwei Ren, Dou Du, Huixiong Xu, Wenwen Yue, Liping Sun

**Affiliations:** ^1^ Department of Medical Ultrasound Shanghai Tenth People's Hospital Ultrasound Research and Education Institute School of Medicine Tongji University Shanghai P. R. China; ^2^ Department of Ultrasound Institute of Ultrasound in Medicine and Engineering Zhongshan Hospital Fudan University Shanghai P. R. China; ^3^ Department of In‐patient Ultrasound the Second Affiliated Hospital Harbin Medical University Harbin P. R. China; ^4^ Department of Medical Ultrasound Henan Provincial People's Hospital School of Medicine Henan University Zhengzhou Henan P. R. China

**Keywords:** CD155/TIGIT blockade, engineered membrane vesicles, genetic engineering, liver metastases, microwave ablation

## Abstract

Microwave ablation (MWA) represents a highly effective and clinically significant therapeutic modality for the treatment of liver metastases. The proliferation of disseminated tumor cells within the peri‐necrotic transition zone (TZ) is a critical factor contributing to the postablative recurrence; to date, no effective method has been identified to specifically target and eliminate these cells. Here, based on an experimental liver metastases model, we leverage single‐cell RNA sequencing and flow cytometry analysis, which reveals that TZ exhibits a VEGF‐mediated immunosuppressive microenvironment, characterized by a significant increase of CD155^+^ myeloid cells. We further report the development of engineered cell membrane vesicles encapsulating Bevacizumab, which are fused with TIGIT‐expressing membranes and platelet membranes (referred to as Bev@TPNVs). The Bev@TPNVs can specifically target the liver and the TZ, inhibit neovascularization, and restore the anti‐tumor functionality of CD8^+^ T cells. Our findings demonstrate that Bev@TPNVs can effectively suppress liver metastasis after MWA. The intrahepatic metastasis burden is reduced by approximately 10‐fold compared with the control group, and the survival rate of mice within 70 days reaches 50%. This work has the potential to establish a novel standard treatment paradigm that could revolutionize combined immunotherapy following liver metastasis ablation.

## Graphical abstract

Microwave ablation (MWA) represents a highly effective and clinically significant therapeutic modality for the treatment of liver metastases. This study develops engineered cell membrane vesicles to specifically target the transition zone of MWA for liver metastases. This strategy can inhibit VEGF‐mediated immunosuppression, block CD155/TIGIT signaling to reinstate CD8^+^ T cell anti‐tumor function, and markedly suppress liver metastases recurrence following MWA.

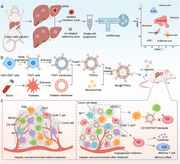

## 1 Introduction

Tumor metastasis is a major contributor to cancer‐related mortality and presents a considerable challenge to the long‐term survival of patients [1, 2]. The liver, being the largest solid organ in the human body, is a frequently affected site for distant metastasis, with up to 50% of patients across various cancer types, such as colorectal cancer, either presenting with or developing liver metastases over the course of their disease [3, 4]. The prognosis of many patients with liver metastases can be poor, with a near‐zero 5‐year survival if the metastases remain untreated [5, 6]. Therefore, achieving effective local control of liver metastases is crucial for improving long‐term prognosis.

Surgical resection is the first‐line therapy recommended by guidelines for liver metastases. However, despite recent advancements in neoadjuvant chemotherapy, surgical resection remains feasible for only 15% to 30% of patients [3]. Local ablation therapies, such as radiofrequency ablation and microwave ablation (MWA), are extensively utilized in the treatment of unresectable liver metastases, a practice supported by a wealth of global clinical experience and international clinical guidelines [7, 8]. Nevertheless, the major limitation of these ablation techniques lies in their relatively high rate of local tumor recurrence, particularly in the transition zone (TZ)—a narrow region of liver tissue surrounding the ablated necrotic core. Clinical studies have shown that the recurrence rate in the TZ is approximately 9% to 18% under conventional conditions [9, 10]. For patients with large tumors (> 3 cm), this recurrence rate can even soar to as high as 60% [11].

Notably, to achieve complete ablation, clinical practice typically involves extending ablation to 0.5 cm of normal liver tissue around the liver metastases to ensure a safe ablation margin. However, a growing body of evidence indicates that this thermal ablation‐induced stimulation of normal liver tissue conversely promotes the colonization and proliferation of disseminated tumor cells within the TZ, directly impairing the efficacy of ablation [12]. Although thermal ablation therapy has been proven to promote tumor growth within the TZ [12], targeted interventions addressing the unique microenvironment of the TZ remain lacking. This research gap highlights two urgent needs: (1) to elucidate the kinetic characteristics and molecular mechanisms underlying ablation‐induced tumor recurrence within the TZ, and (2) to develop intervention strategies that can precisely target the TZ, reverse the tumor‐promoting state within the TZ, and inhibit recurrence of liver metastases after ablation.

Based on these understandings, we constructed a preclinical colorectal liver metastases (CRLM) model via hemi‐splenic injection and found that the survival duration of mice with CRLM was significantly shortened following MWA stimulation. From the third day after MWA, the expression of tumorigenic factors, such as vascular endothelial growth factor (VEGF), was elevated within the TZ, accompanied by increased tumor cell proliferation and neovascularization. Further, high‐dimensional single‐cell RNA sequencing (scRNA‐seq) analysis and flow cytometry (FCM) data from TZ tissues on day 3 post‐ablation indicated that the TZ presented an immunosuppressive microenvironment characterized by high levels of CD155^+^ myeloid cells mediated by VEGF. CD155 binds to the T‐cell immunoreceptor with immunoglobulin and immunoreceptor tyrosine‐based inhibition motif domain (TIGIT), thereby facilitating immune evasion [13]. These findings indicate that a rational combination of anti‐VEGF therapy and CD155/TIGIT blockade can effectively enhance the therapeutic efficacy of local ablation treatment for liver metastases.

**FIGURE 1:**
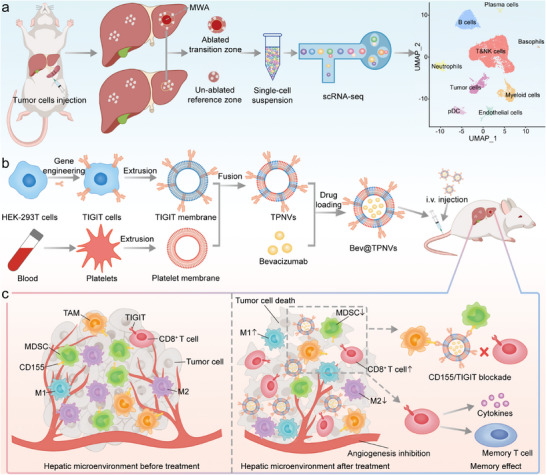
Schematic of Bev@TPNVs synergizing with microwave ablation (MWA) to mediate liver metastases eradication. (a) Construction of liver metastasis MWA model via hemi‐splenic injection and high‐dimensional single‐cell RNA sequencing (scRNA‐seq) analysis of the ablated transition zone (TZ). (b) Schematic for the preparation of Bev@TPNVs. (c) Schematic diagram of the effects of Bev@TPNVs on liver metastasis after MWA, including inhibiting neovascularization and infiltration of immunosuppressive cells, enhancing the immune response of CD8^+^ T cells, and eliciting systemic immune memory effects. i.v., intravenous; MDSC, myeloid‐derived suppressor cell; TAM, tumor‐associated macrophage; M1, M1‐like macrophage; M2, M2‐like macrophage.

Here, we developed engineered cell membrane vesicles (Bev@TPNVs) to enhance the response to combined anti‐VEGF therapy with CD155/TIGIT blockade against tumor recurrence after MWA of CRLM. Cell membrane‐derived nanovesicles (CMVs) exhibit promising translational potential in cancer therapy, owing to their excellent biocompatibility and minimal toxicity profiles [14]. However, the inherent heterogeneity of CMVs poses challenges in achieving precise therapeutic functions, such as organ‐specific targeting and signal regulation [15]. Recent advancements suggest that the modification of CMVs through genetic engineering, ultrasound, and other techniques can significantly enhance their therapeutic specificity and functionality [15, 16]. Consequently, the development of multifunctional hybrid cell membrane vesicles that allow for achieving accurate regulation and targeting postoperative wounds holds immense significance in advancing targeted therapies post‐ablation. In our study, Bev@TPNVs were developed by merging engineered TIGIT cell membrane nanovesicles (TNVs) and platelet membrane nanovesicles (PNVs), thereby enabling the encapsulation of Bevacizumab (a monoclonal antibody against VEGF) (Figure 1). PNVs specifically target wound sites following liver ablation, while TIGIT from TNVs competes with CD155 on myeloid cells for binding, thereby blocking the CD155/TIGIT pathway and restoring the anti‐tumor immune response mediated by CD8^+^ T cells. Additionally, Bevacizumab collaborates with TNVs to decrease myeloid‐derived suppressor cells (MDSCs) and facilitates the polarization of tumor‐associated macrophages (TAMs) into M1 phenotypes. Our findings demonstrate that Bev@TPNVs achieve a ∼10‐fold reduction in intrahepatic metastasis burden compared with the control group, and the survival rate of mice reaches 50% within 70 days. This study represents the first investigation to provide valuable insights into the kinetics and underlying principles governing ablation‐induced tumorigenesis at the single‐cell level. These findings thus support the potential clinical utility of synergistically combining TIGIT checkpoint blockade with anti‐VEGF therapy to prevent tumor recurrence after liver metastasis ablation.

## 2 Results

### 2.1 Focal Hepatic MWA Enhances Perinecrotic Tumor Progression of Colorectal Liver Metastases

**FIGURE 2:**
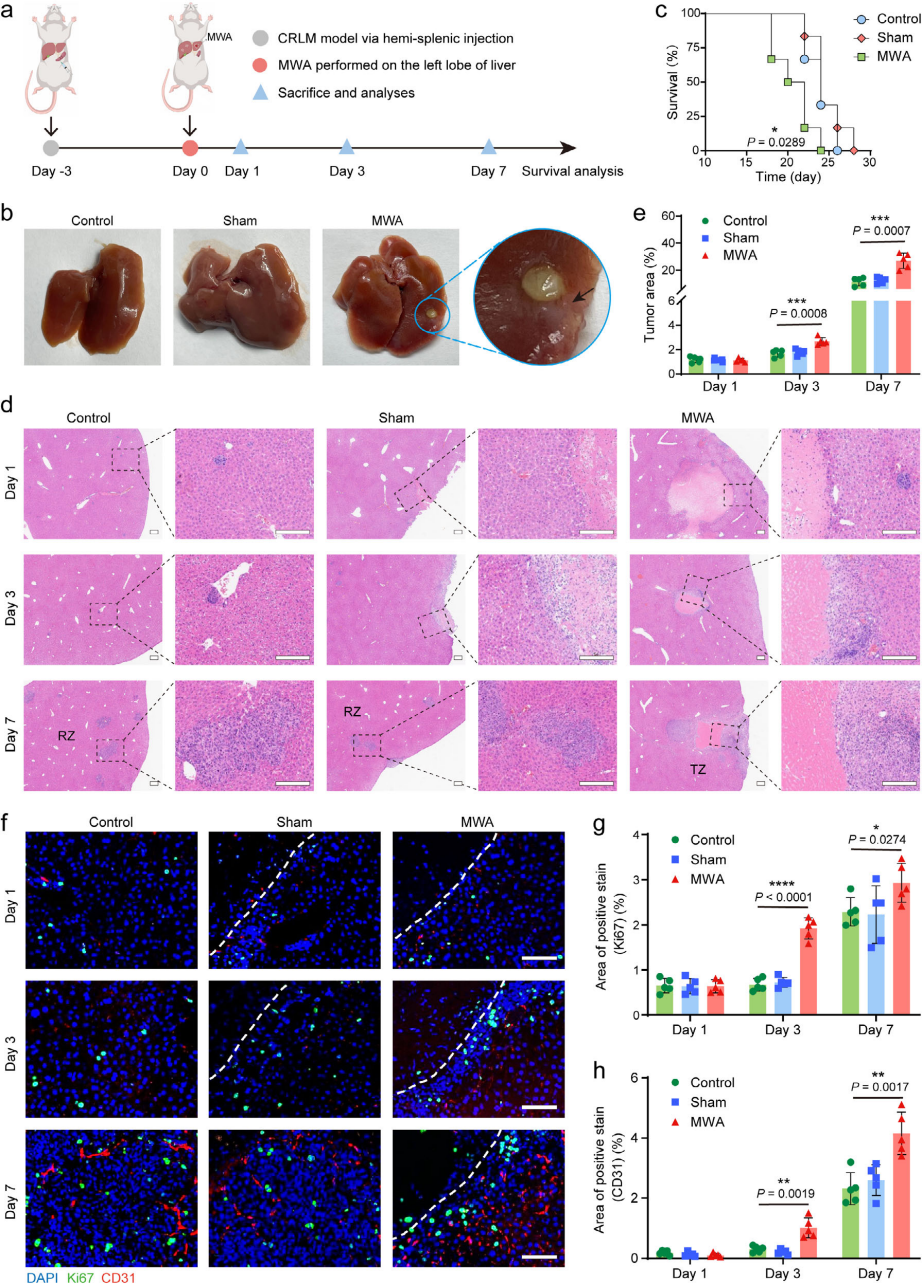
Microwave ablation (MWA) induces ablated transition zone (TZ) tumorigenesis in colorectal liver metastases (CRLM) mouse model. (a) Construction of focal hepatic MWA of the CRLM model. (b) Representative gross images of intrahepatic metastatic burden on day 7 for control, sham operation, and MWA‐stimulated mice. Black arrow indicates liver metastases. (c) Survival curve analysis of various groups after the establishment of the liver metastasis model (*n* = 6 mice per group). Asterisk denotes a significant difference compared with the control and MWA groups. (d) Hematoxylin and eosin (H&E)‐stained sections of mice livers containing metastases in the reference zone (RZ) and in the TZ surrounding the necrotic central area (Scale bars: 200 µm). (e) Tumor burden in the RZ and TZ was quantitatively assessed using images acquired at 4 × magnification. Data are presented as means ± standard deviation (SD) (*n* = 5 biologically independent samples). (f) Representative immunofluorescence images at different time points displaying the infiltration of Ki67 and CD31 in the RZ of the control group and the sham‐operated group, as well as in the TZ of the MWA group (Scale bars: 50 µm). The dotted lines are located at the outer edge of the injury or necrosis. (g), (h) Corresponding quantification of Ki67 and CD31. Data are presented as means ± standard deviation (SD) (*n* = 5 biologically independent samples). Statistical significance was calculated using the log‐rank (Mantel‐Cox) test for c or two‐tailed unpaired Student's *t*‐test for e, g, and h. ^*^ *p* < 0.05, ^**^ *p* < 0.01, ^***^ *p* < 0.001, and ^****^ *p* < 0.0001.

To investigate the effect of focal hepatic MWA on the outgrowth of diffuse residual cancer cells in the liver, an aggressive CRLM model was established through hemi‐splenic injection (Figure 2a). Compared to the control and sham ablation groups, noticeable tumor invasion was observed in the TZ adjacent to the necrotic zone on the seventh day post‐ablation (Figure 2b). The survival time of sham‐operated and untreated mice exhibited no significant difference, whereas that of MWA‐treated mice was substantially reduced (Figure 2c). These findings indicated that MWA stimulation potentially accelerates the progression of diffuse metastatic disease in the TZ adjacent to the necrotic area.

To further explore the kinetics and underlying pathological characteristics responsible for the worsened survival outcomes following thermal ablation stimulation, liver samples were collected on the 1st, 3rd, and 7th days after different treatments, and hematoxylin‐eosin (H&E) staining was performed to observe the tumor proliferation in the ablated TZ and the unablated reference zone (RZ). The definition of the TZ was based on the histological data, showing TZ characteristics being most abundant within a 2‐mm range extending outward from the edge of necrosis. The RZ was designated as the remaining part of the liver. Notably, the tumor burden in TZ exhibited a significant increase starting from the third day in the MWA group, and by the seventh day, it had increased 2‐ to 3‐fold compared to that in the RZ of control mice (26.94% ± 5.66% vs. 11.46% ± 3.05%, *P* = 0.0007) (Figure 2d, e). The tumor proliferation and neovascular infiltration were also assessed through immunofluorescence analysis. Our findings revealed no statistically significant differences in either research indicator one day after MWA (Figures 2f–h; S1). However, on days 3 and 7, the proportion of Ki67‐positive cells in the TZ of the MWA group was significantly higher than that in the RZ of the untreated and sham‐operated groups, concomitant with an increase in CD31‐stained microvessels (Figure 2f–h). Collectively, these data suggested that the ablated TZ displayed an elevated proliferation index and enhanced neovascularization, with a distinct expression window on the third day following ablation, thereby contributing to the progression of liver metastasis.

### 2.2 Single‐Cell Transcriptome Atlas of TZ Liver Tissue Reveals Distinctive Characteristics of the Immunosuppressive Microenvironment

**FIGURE 3:**
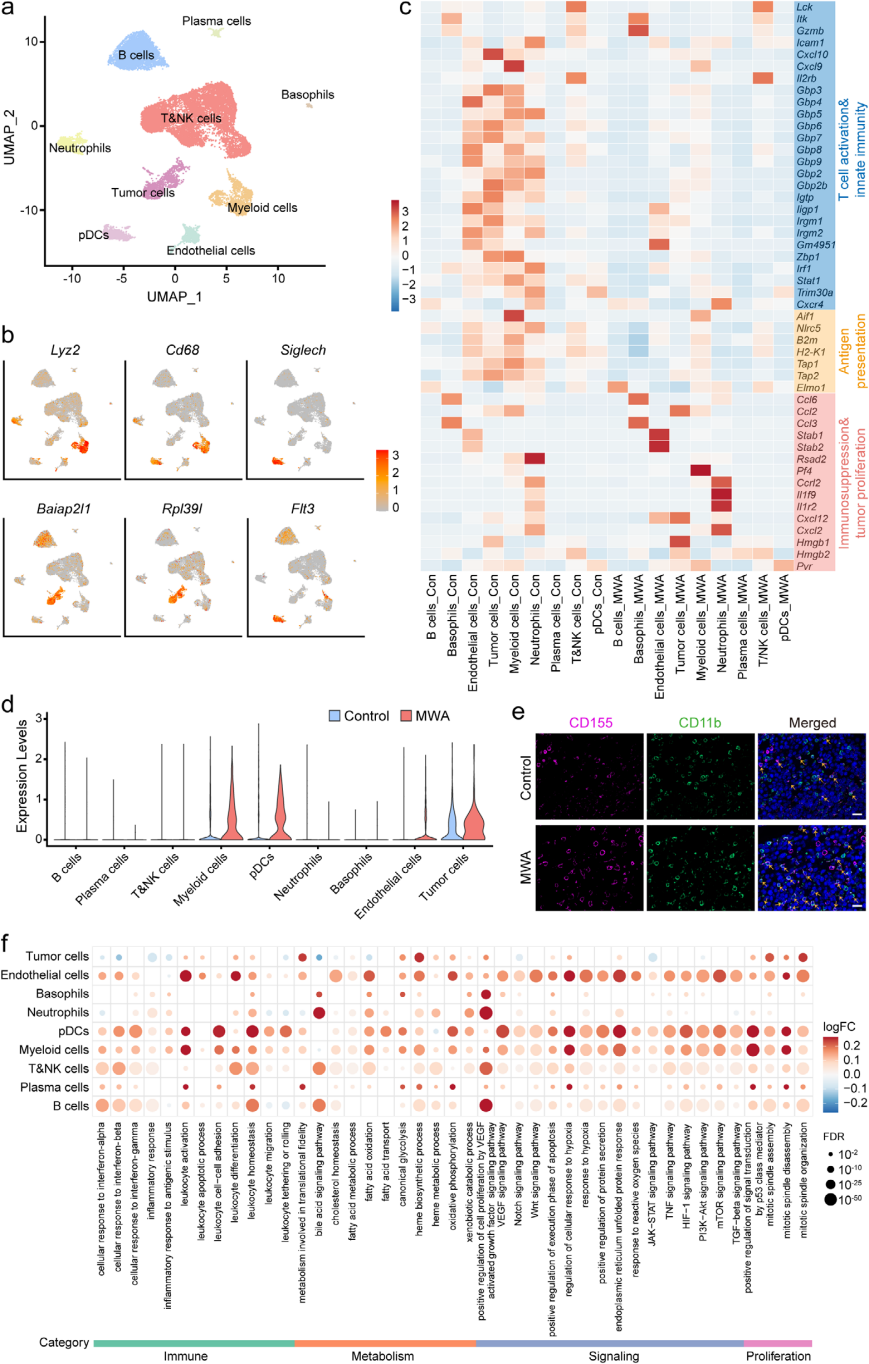
Single‐cell atlas and immunofluorescence staining analysis of tissues in the control and ablated transition zone (TZ) of liver metastasis. (a) UMAP plots of cells from two groups aggregated, with 9 clusters identified in each plot. Each cluster was represented by a different color. (b) Expression levels of selected classical marker genes in unsorted cells are shown in UMAP plots from both control and ablated TZ tissues in liver metastasis mice. (c) Expression in each cluster of immune response‐related genes in liver metastases before and after microwave ablation (MWA). (d) Violin plots showing the probability distribution of CD155 expression across different cell clusters. (e) Representative immunofluorescence images of liver tissues of the control group and the TZ after MWA stimulation (Scale bars: 20 µm). (f) Dot plots of hallmarks for differential pathways in the overall cell types between tissues in the pre‐MWA and ablated TZ. The intensity of the dots indicates the average fold change of pathway expression in liver metastases after MWA compared with that before MWA. The dot size represents the false discovery rate (FDR) for each hallmark.

To obtain an objective understanding of the underlying molecular mechanisms responsible for the protumorigenic effects observed in TZ stimulated by MWA of liver metastases, we collected TZ liver tissue and micrometastasis control liver tissue 3 days post‐ablation. The samples were promptly processed using the 10 × Genomics platform for 3'‐end scRNA‐seq (Figure 1a). A total of 14,762 cells were examined in the control group, while 10,247 cells were analyzed in the MWA group. Quality control was passed by 86.55% of cells in the control group and 93.47% in the MWA group. The median number of genes per cell was 2,049 and 2,526 for the control and MWA groups, respectively. Based on the expression of classical markers, a total of 22,357 cells were integrated, segmented, and classified into nine cell populations (Figures 3a, b; S2 and S3), including T&NK (T and natural killer) cells identified by the expression of *Cd3e* and *Cd3g* [17], B cells expressing *Ms4a1* and *Cd79a* [18], myeloid cells marked by *Lyz2* and *CD68* [19], CT26 tumor cells are positive for *Baiap2l1* and *Rpl39l* expression [20], endothelial cells defined by their classical markers *Cdh5* and *Flt1* [18], neutrophils which expressed *S100a8* and *S100a9* [21], plasmacytoid dendritic cells (pDCs) marked by *Siglech* and *Flt3* [22], plasma cells identified by *Jchain* and *Mzb1* expression [23], and basophils marked by *Gata2* and *Ms4a2* [24].

In addition, we found that genes involved in T cell activation, innate immunity, and antigen presentation were downregulated in the ablated TZ, while genes associated with immune suppression and tumor proliferation were upregulated (Figure 3c). These findings indicated that MWA induced immunosuppression in the liver microenvironment within the TZ. Notably, the expression of CD155 (encoded by the *Pvr* gene), a tumor proliferation‐related gene, was upregulated in the MWA group (Figure 3c). Additionally, violin plots revealed high CD155 expression in myeloid cells and pDCs within the TZ (Figure 3d). Immunofluorescence staining further demonstrated a significant increase in the colocalization of CD155 with CD11b^+^ myeloid cells in the TZ of the MWA group compared to the control group (Figure 3e). Moreover, data in Table S1 showed that the proportions of CD155^+^ myeloid cells and CD155^+^ pDCs in the MWA group were significantly elevated, reaching 1.95‐fold and 2.19‐fold those of the control group, respectively. This suggested that MWA effectively promoted the expansion of CD155^+^ subpopulations in these two cell types. Further analysis of expression intensity revealed that the mean unique molecular identifier (UMI) counts of the *Pvr* gene in CD155^+^ myeloid cells and CD155^+^ pDCs were higher in the MWA group (2.184615 and 2.431694, respectively) than in the control group (1.749288 and 1.65, respectively) (Table S1). Collectively, these results indicated that MWA not only increased the proportion of CD155^+^ cells but also upregulated CD155 expression in individual CD155^+^ cells. These two effects may synergistically enhance CD155‐mediated immunomodulatory effects in the TZ microenvironment.

In order to investigate alterations in the regulatory network of ablated TZ‐infiltrating cell subsets, we utilized the signature gene sets from the Molecular Signature Database (MsigDB) to analyze pathway changes in each cell subset between liver tissues in the control and ablation groups (Figure 3f). The expression levels of metabolism‐related genes (e.g., *Apoe*, *Abca1*, *Srebf2*) and proliferation‐related genes (e.g., *Tacc1*, *Kif2a*, *Pibf1*) in TZ‐infiltrating myeloid cells, pDCs, and endothelial cells were significantly higher than those from liver tissue without ablation (Figures 3f; S4). These findings indicate that immunometabolism was reprogrammed in post‐ablated liver metastases. In addition, immune‐related pathways, PI3K‐Akt signaling pathways, and VEGF signaling pathways were enriched in both myeloid cells and pDCs in the ablated TZ compared to control liver tissue (Figure 3f), suggesting myeloid cells were involved in the immune response post‐ablation in liver metastases. Previous studies have demonstrated that the activation of the VEGF pathway associated with angiogenesis can mediate the activation of downstream pathways such as the PI3K‐Akt pathway, resulting in increased production of inflammatory cytokines and enhanced migration of myeloid cells [25, 26]. Based on these observations, we hypothesize that a VEGF‐mediated regulatory pathway for myeloid cells is established in the hepatic microenvironment of CRLM following MWA. This pathway may lay the foundation for the subsequent formation of an immunosuppressive microenvironment.

### 2.3 The Critical Role of CD155^+^ Myeloid Cells in MWA‐Induced Immunosuppression of the TZ

**FIGURE 4:**
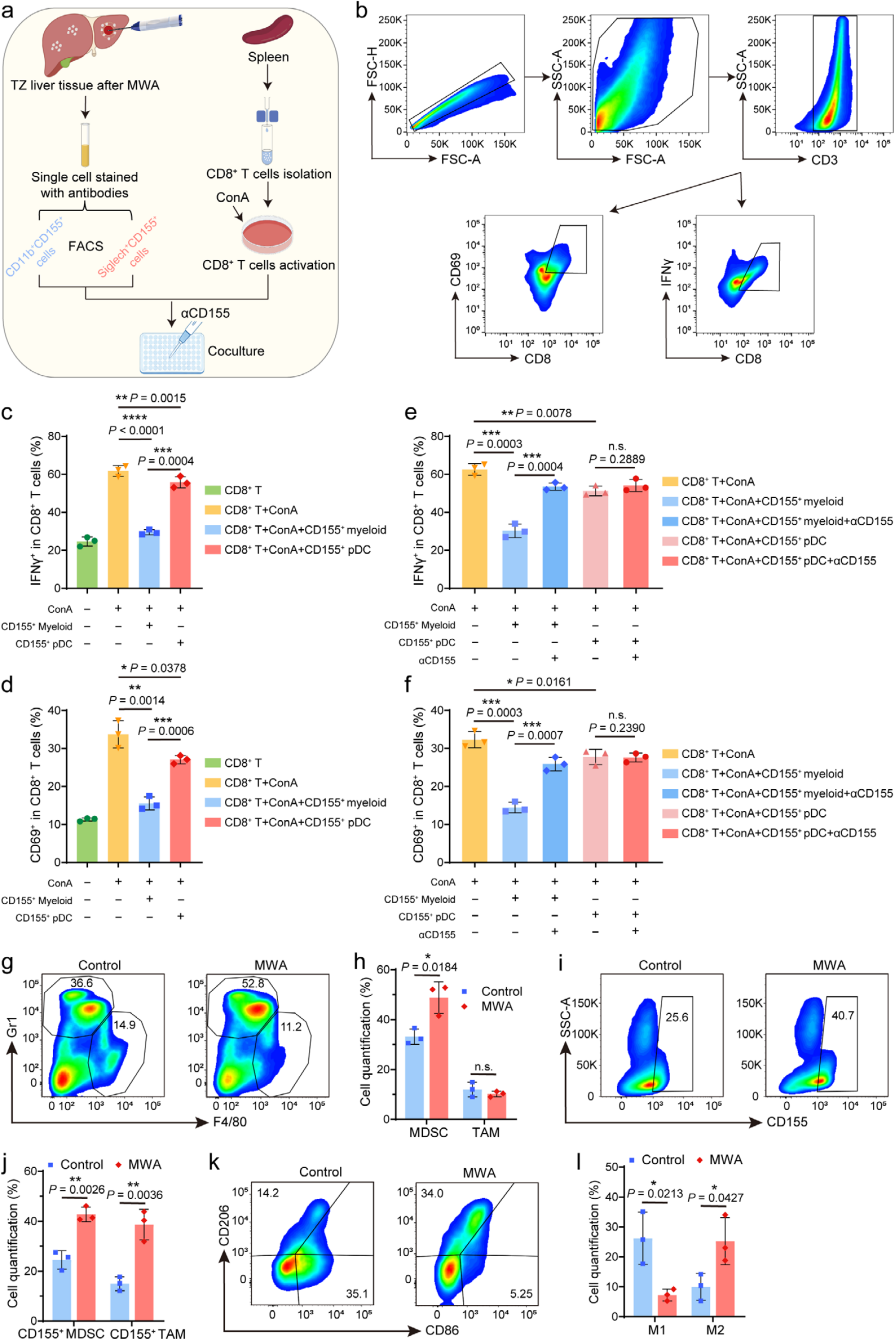
Verification of the immunosuppression of CD155^+^ myeloid cells in the TZ induced by MWA. (a) Schematic illustration of the CD8^+^ T cell co‐culture inhibition assay. (b) Gating scheme for analyzing IFN‐γ^+^CD8^+^ T and CD69^+^CD8^+^ T cells of the co‐cultured cells. (c–f) Quantification of flow cytometry (FCM) analysis of IFN‐γ^+^CD8^+^ T and CD69^+^CD8^+^ T cells. Data are presented as means ± SD (*n* = 3 biologically independent samples). (g), (h) Representative FCM analysis (g) and corresponding quantification (h) of myeloid‐derived suppressor cells (MDSCs, CD11b^+^Gr1^+^) and tumor‐associated macrophages (TAMs, CD11b^+^F4/80^+^) in CD45^+^ cells. Data are presented as means ± SD (*n* = 3 biologically independent samples). (i, j) Representative FCM analysis of CD155^+^ MDSCs (CD11b^+^Gr1^+^CD155^+^) in CD45^+^ cells (i) and quantification of CD155^+^ MDSCs and CD155^+^ TAMs (CD11b^+^F4/80^+^CD155^+^) in CD45^+^ cells (j). Data are presented as means ± SD (*n* = 3 biologically independent samples). (k), (l) Representative FCM analysis (k) and corresponding quantification (l) of TAMs‐M1 (CD86^+^) and TAMs‐M2 (CD206^+^) in CD11b^+^F4/80^+^ cells. Data are presented as means ± SD (*n* = 3 biologically independent samples). Statistical significance was calculated using a two‐tailed unpaired Student's *t*‐test. n.s., not significant, ^*^ *p* < 0.05, ^**^ *p* < 0.01, ^***^ *p* < 0.001, and ^****^ *p* < 0.0001.

To further clarify the specific role of myeloid cells in immunosuppression and the key signaling pathways involved, we performed functional validation focusing on CD155^+^ cell subsets. CD155^+^ myeloid cells (CD11b^+^CD155^+^) and CD155^+^ pDCs (Siglech^+^CD155^+^) were isolated from TZ tissues post‐MWA via fluorescence‐activated cell sorting (FACS). Concurrently, CD8^+^ T cells were purified from mouse spleens using magnetic‐activated cell separation (MACS) (Figure 4a). After co‐culturing concanavalin A (ConA)‐activated CD8^+^ T cells with each of the two CD155^+^ cell subsets separately, FCM analysis revealed that CD155^+^ myeloid cells exerted a significantly stronger inhibitory effect on CD69 expression and interferon‐γ (IFN‐γ) secretion in CD8^+^ T cells compared to CD155^+^ pDCs (Figure 4b–d). To further validate the mechanism, pathway blockade experiments were conducted. The results showed that the inhibitory effect of CD155^+^ myeloid cells on CD8^+^ T cells was markedly reversed upon the addition of an anti‐CD155 antibody (αCD155). In contrast, αCD155 failed to alter the weak inhibitory effect of CD155^+^ pDCs on CD8^+^ T cells (Figure 4e, f). Notably, studies have demonstrated that CD155 can bind to TIGIT molecules on the surface of T cells, thereby inhibiting T cell activation and promoting tumor immune evasion [27]. Collectively, these findings demonstrated that in the MWA‐induced immunosuppressive microenvironment of the TZ, CD155^+^ myeloid cells were the primary cell type mediating immunosuppression, and their inhibitory effect on CD8^+^ T cell activation was dependent on the CD155/TIGIT signaling pathway. To elucidate the pivotal regulatory role of VEGF in CD155^+^ myeloid cells after MWA, Bevacizumab (Bev) was used to deplete VEGF, and an anti‐IgG antibody was used as a control (Figure S5a). Compared with mice treated with the IgG isotype control antibody, the proportions of myeloid cells and CD155^+^ myeloid cells in the TZ of mice treated with Bev were significantly reduced (Figure S5b–e).

Next, we further characterized the specific subsets and phenotypic features of CD155^+^ myeloid cells in the control and the MWA group. Myeloid cells mainly include myeloid‐derived suppressor cells (MDSCs, CD11b^+^Gr1^+^) and TAMs (CD11b^+^F4/80^+^), and these cells play a crucial role in the immunogenicity and progression of cancers [28]. FCM analysis revealed that, in comparison to the control group, the MWA group exhibited a significantly higher infiltration of MDSCs and comparable levels of TAMs (Figures 4g, h; S6). Notably, the numbers of CD155^+^MDSCs and CD155^+^TAMs were significantly elevated in the ablation group (Figures 4i,j; S7), suggesting that these two subsets may synergistically contribute to immunosuppression through high CD155 expression. TAMs are known for their functional heterogeneity, with M1‐like macrophages (TAMs‐M1, CD11b^+^F4/80^+^CD80^+^) infiltration linked to a favorable prognosis, while M2‐like macrophages (TAMs‐M2, CD11b^+^F4/80^+^CD206^+^) are involved in negative immune regulation and immune evasion [28, 29]. Further analysis of TAM phenotypes revealed that the infiltration of TAMs‐M1 was significantly reduced in the TZ of the MWA group. In contrast, the infiltration of TAMs‐M2 was significantly increased (Figure 4k, l). This further indicated that MWA not only recruited myeloid cells but also enhanced immunosuppressive effects by reshaping their phenotypes. Taken together, our data demonstrated that VEGF mediated the recruitment of myeloid cells (particularly MDSCs and TAMs‐M2) to the TZ. Simultaneously, MWA induced high CD155 expression in these myeloid cells, which then inhibited CD8^+^ T cell activation via the CD155/TIGIT pathway. Therefore, targeting the VEGF pathway with an anti‐VEGF antibody to block myeloid cell recruitment and simultaneously targeting CD155^+^ myeloid cells‐mediated CD155/TIGIT pathway to restore CD8^+^ T cell function, holds promise for reversing the post‐MWA immunosuppressive state in the TZ, and thereby inhibiting the recurrence of liver metastases.

### 2.4 Engineering and Characterization of TPNVs

**FIGURE 5:**
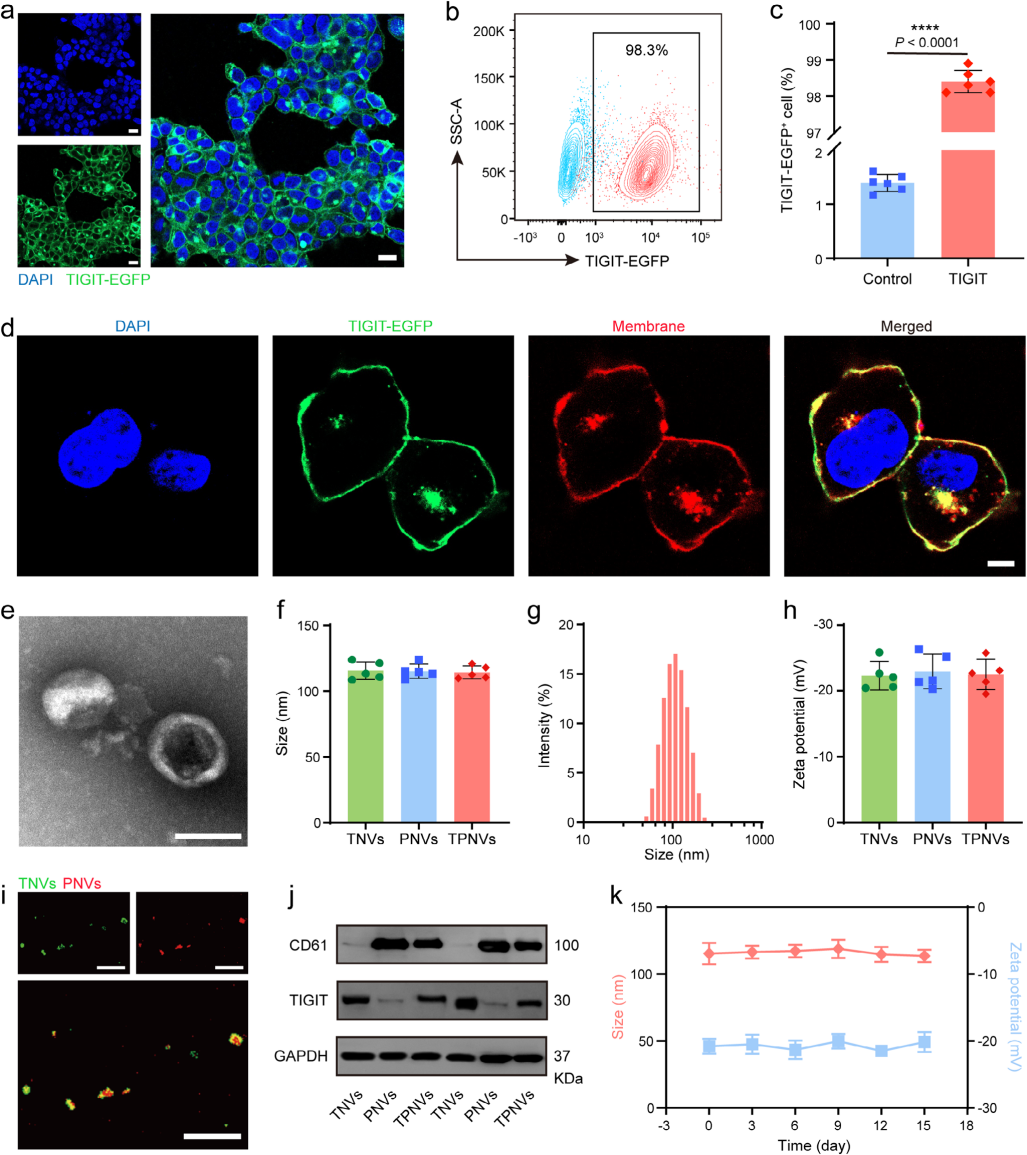
Characterization of TPNVs. (a) Confocal images of TIGIT protein expression on the engineered human embryonic kidney (HEK)‐293T stable cell lines (Scale bars: 20 µm). (b), (c) Representative flow cytometry (FCM) analysis (b) and quantification (c) of TIGIT protein expression on the engineered HEK‐293T cells. Data are presented as means ± SD (*n* = 5 independent samples). (d) Confocal images of TIGIT protein expression on the cell membrane (Scale bar: 5 µm). (e) Transmission electron microscopy (TEM) image of TPNVs (Scale bar: 200 nm). (f) Size of TNVs, PNVs, and TPNVs analyzed by dynamic light scattering (DLS). Data are presented as means ± SD (*n* = 5 independent samples). (g) Particle size distribution of TPNVs measured by DLS. (h) Zeta potential of TNVs, PNVs, and TPNVs analyzed by DLS. Data are presented as means ± SD (*n* = 5 independent samples). (i) Confocal images of TPNVs (Scale bars: 200 µm). (j) Western blot analysis of protein CD61 and TIGIT in the samples of TNVs, PNVs, and TPNVs. (k) Stability assessment of TPNVs. Mean diameter and zeta potential of TPNVs in PBS over 15 days. Data are presented as means ± SD (*n* = 5 independent samples). Statistical significance was calculated using a two‐tailed unpaired Student's *t*‐test, ^****^ *p* < 0.0001.

Given the exceptional biocompatibility and precise targeting capabilities inherent in engineered fused membrane vesicles, we synthesized TPNVs. To obtain and characterize TIGIT‐containing cell membranes, we first infected human embryonic kidney (HEK)‐293T cells with a lentivirus encoding the TIGIT gene fused with an enhanced green fluorescent protein (EGFP) tag. Stably TIGIT‐EGFP‐expressing cells were then selected using puromycin. Confocal laser scanning microscopy (CLSM) revealed prominent green fluorescence on nearly all cells, confirming successful synthesis of engineered TIGIT cells (Figure 5a; Figure S8). Flow cytometry analysis indicated that more than 98% of cells were TIGIT‐EGFP^+^ (Figures 5b, c; S9). The co‐localization of the cell membrane dye 1,1'‐octadecyl‐3,3,3',3'‐tetramethylindocyanine perchlorate (Dil) and EGFP demonstrated that TIGIT was primarily localized to the cell membrane (Figures 5d; S10).

The cell membranes of TIGIT‐EGFP cells were extracted to prepare TNVs, and platelets were isolated from mouse blood to prepare PNVs (Figure S11). Subsequently, the obtained TNVs and PNVs were combined, sonicated, and extruded repeatedly to generate TPNVs. Transmission electron microscopy (TEM) and dynamic light scattering (DLS) analysis revealed that TPNVs were elliptical in shape with a diameter of approximately 110 nm (Figure 5e–g). Zeta potential measurements indicated that the potential of TPNVs was approximately −22 mV, which was not significantly different from that of TNVs and PNVs (Figure 5h). TNVs and PNVs labeled with different fluorescent dyes displayed clear overlap of fluorescence signals under CLSM, confirming the successful formation of TPNVs through the fusion of TNVs and PNVs (Figure 5i). Additionally, western blot analysis further demonstrated the presence of specific protein markers in TPNVs, including TIGIT and the platelet‐specific marker CD61 (Figure 5j). Moreover, TPNVs demonstrated good stability, as indicated by their ability to be stored in PBS for a minimum of two weeks (Figure 5k).

### 2.5 In vitro Biological Effects and In vivo Perinecrotic Targeting of Bev@TPNVs

**FIGURE 6:**
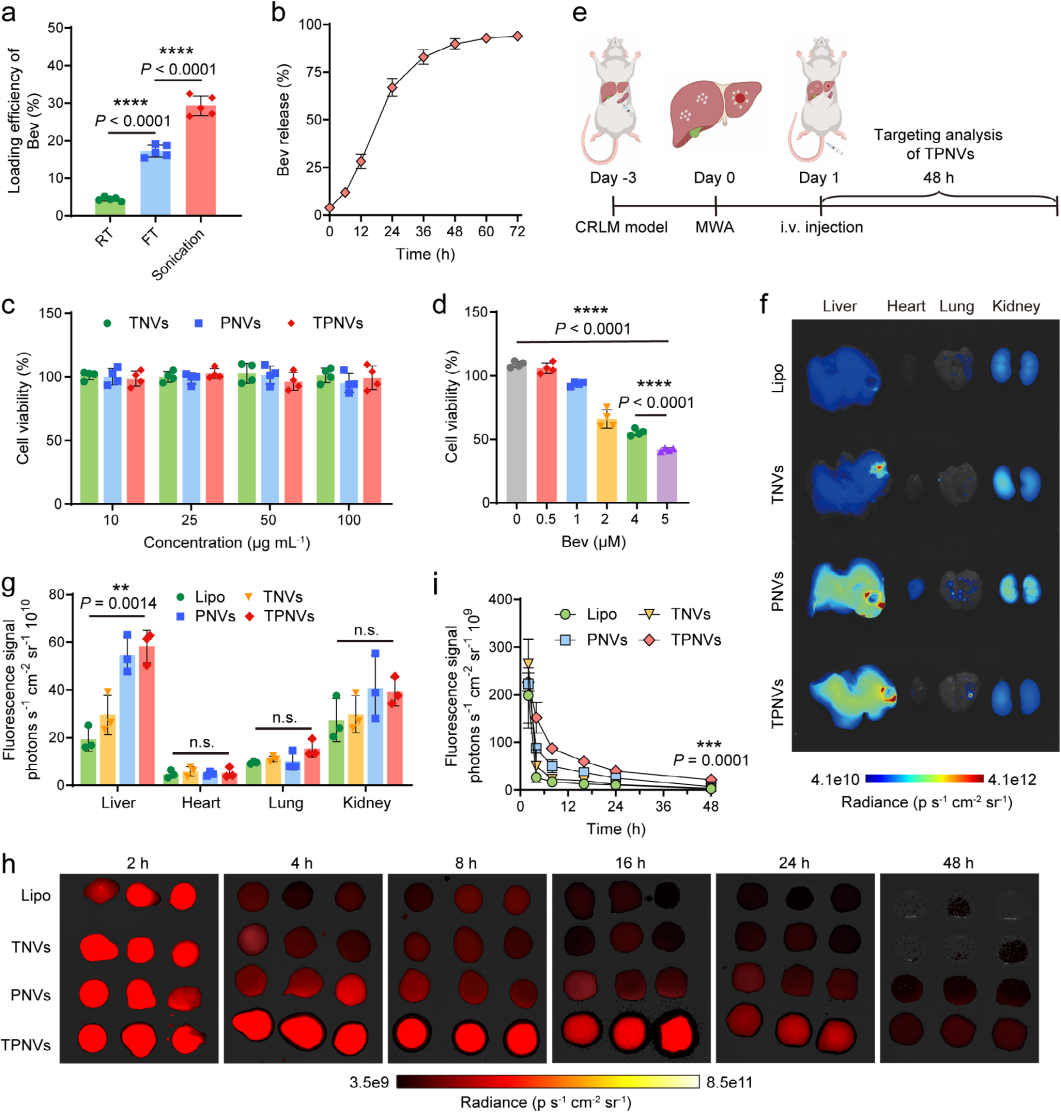
The biological effects of Bev@TPNVs and the capacity to reduce the accelerated tumor progression following MWA. (a) Loading efficiency of Bev in TPNVs under different treatment conditions, including room temperature (RT), freeze‐thaw cycles (FT), and sonication. Data are presented as means ± SD (*n* = 5 independent samples). (b) In vitro Bev release profile from TPNVs. Data are presented as means ± SD (*n* = 5 independent samples). (c) In vitro biocompatibility of TNVs, PNVs, and TPNVs. Data are presented as means ± SD (*n* = 4 independent samples). (d) Detection of tumor cell viability after Bev treatment. Data are presented as means ± SD (*n* = 4 independent samples). (e) Schematic showing the targeting analysis of TPNVs after liver metastasis ablation. (f), (g) Fluorescence imaging (f) and quantitative analysis (g) of liposomes, TNVs, PNVs, and TPNVs distributed in major organs, including liver, heart, lung, and kidney. Data are presented as means ± SD (*n* = 3 biologically independent samples). (h) Fluorescence imaging of liposomes, TNVs, PNVs, and TPNVs distributed in blood after different times post‐injection. (i) In vivo pharmacokinetic curves of liposomes (Lipo), TNVs, PNVs, and TPNVs. Data are presented as means ± SD (*n* = 3 biologically independent samples). Asterisk denotes a significant difference compared with the Lipo and TPNVs groups. Statistical significance was calculated using a two‐tailed unpaired Student's *t*‐test. n.s., not significant, ^**^ *p* < 0.01, ^***^ *p* < 0.001, and ^****^ *p* < 0.0001.

To achieve efficient drug loading efficiency (DLE), we used three different methods to load Bev into TPNVs. Our findings revealed that incubation at room temperature (RT) resulted in the poorest DLE for Bev. Among the tested methods, sonication outperformed freeze‐thaw (FT) cycle incubation, achieving the highest DLE of approximately 29.26% for Bev (Figure 6a). The kinetics of Bev release from TPNVs indicated that the cumulative release percentage of Bev increased over time, with approximately 93.96% released within three days (Figure 6b). TPNVs demonstrated no obvious toxicity, and a certain concentration of Bev@TPNVs effectively inhibited the growth of CT26 tumor cells (Figure 6c, d). Platelet‐derived NVs, characterized by a phospholipid bilayer structure, exhibited prolonged systemic circulation time and the ability to target damaged vasculature and postoperative wounds [30, 31]. To assess the targeting ability of TPNVs, a mouse model of CT26 liver metastasis was utilized to evaluate the pharmacokinetics and tissue distribution of various NVs administered via tail vein post‐hepatic MWA (Figure 6e). Cy5.5‐NHS‐labeled liposomes (Lipo), TNVs, PNVs, and TPNVs were injected into mice one day after ablation. As depicted in Figure 6f, g, organ fluorescence imaging at 48 h indicated significant accumulation of TPNVs in the liver, particularly within the ablated TZ. Furthermore, PNVs and TPNVs displayed sustained presence in the blood, with detectable fluorescence 48 h post‐injection, which was facilitated by platelet‐derived targeting moieties and TIGIT on TPNVs (Figure 6h, i). Collectively, these data suggested that TPNVs were capable of efficiently loading and releasing Bev. Moreover, they could specifically target the TZ following liver ablation.

### 2.6 Bev@TPNVs Reduce the Accelerated Tumor Progression Following MWA

**FIGURE 7:**
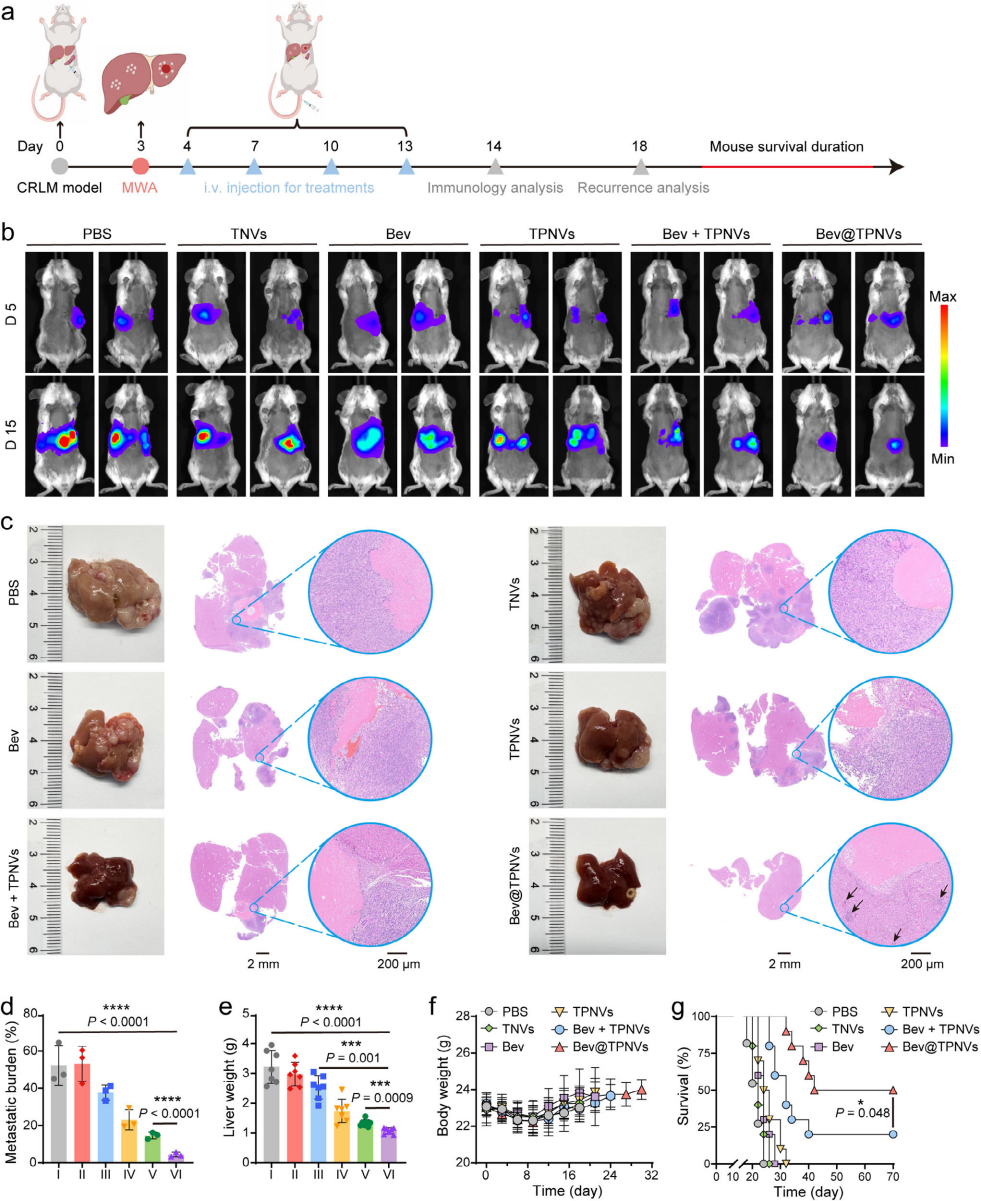
The biological effects of Bev@TPNVs and their capacity to reduce the accelerated tumor progression following MWA. (a) Schematic illustration of different treatment protocols in the mouse model of liver metastasis ablation. (b) In vivo bioluminescence imaging of mice receiving various treatments after liver metastasis ablation. (c) Representative gross images of mouse livers and corresponding hematoxylin and eosin (H&E) staining sections. (d), (e) Quantification of liver metastatic burden (d) (*n* = 3 biologically independent samples) and weight (e) (*n* = 7 biologically independent samples) post different treatments. (f) Body weights of mice in various treatment groups (*n* = 10 mice per group). (g) Kaplan‐Meier survival curves for mice as indicated (*n* = 10 mice per group). All data are presented as mean ± SD. Statistical significance was calculated using a two‐tailed unpaired Student's *t*‐test for d and e or log‐rank (Mantel‐Cox) test for g. ^*^ *p* < 0.05, ^***^ *p* < 0.001, and ^****^ *p* < 0.0001.

Having confirmed that Bev@TPNVs could accumulate in the TZ subsequent to hepatic MWA, we speculated that alterations in the hepatic immune microenvironment mediated by Bev@TPNVs may impede the progression of hepatic metastasis following MWA. In a Luc‐CT26 liver metastasis model, mice underwent MWA on the third day, followed by treatment with PBS, TNVs, Bev, TPNVs, free Bev + TPNVs, or Bev@TPNVs on days 4, 7, 10, and 13 as per the experimental protocol (Figure 7a). Liver metastasis was tracked using bioluminescent signals (Figure 7b). Following four treatment cycles, the metastatic burden was assessed on day 18 through H&E staining of liver tissue sections and liver weight measurements to evaluate the therapeutic efficacy of Bev@TPNVs on liver metastases post‐ablation. The dissected livers are depicted in Figure S12. The results revealed that the PBS group exhibited a higher burden of liver metastasis compared to the other treatment groups, especially with notable tumor invasion at the ablated TZ (Figures 7c; S12). Bev and TPNVs exhibited limited inhibitory effects on liver metastasis compared to the Bev@TPNVs group. Excitingly, H&E staining of livers from mice treated with Bev@TPNVs showed minimal punctate metastases in the liver, with a metastatic burden approximately 10‐fold lower than that in the PBS group, accompanied by a 2‐fold reduction in liver weight (Figure 7d, e). Furthermore, post‐hoc power analysis (G*Power 3.1) revealed that the Cohen's d values for all the aforementioned core comparisons exceeded 5, with statistical power (1‐β) greater than 0.97, which strongly corroborates the robustness of the above findings. Additionally, the body weight of mice in all groups remained stable throughout the study (Figure 7f). Mice treated with Bev@TPNVs showed reduced circulating levels of aspartate transaminase (AST) and alanine transaminase (ALT) compared to the PBS group, indicating that Bev@TPNVs improved liver function while alleviating hepatic tumor burden (Figure S13). To monitor the survival rate of mice, the liver metastasis ablation model was constructed as per the aforementioned procedures, and mice were observed for up to 70 days. Mice in the PBS, TNVs, Bev, and TPNVs groups all died within 40 days (Figure 7g). Notably, mice treated with Bev@TPNVs exhibited the longest survival time, with a 50% survival rate on day 70.

### 2.7 Bev@TPNVs Trigger Robust Antitumor Immune Response and Systemic Immune Memory Effect

**FIGURE 8:**
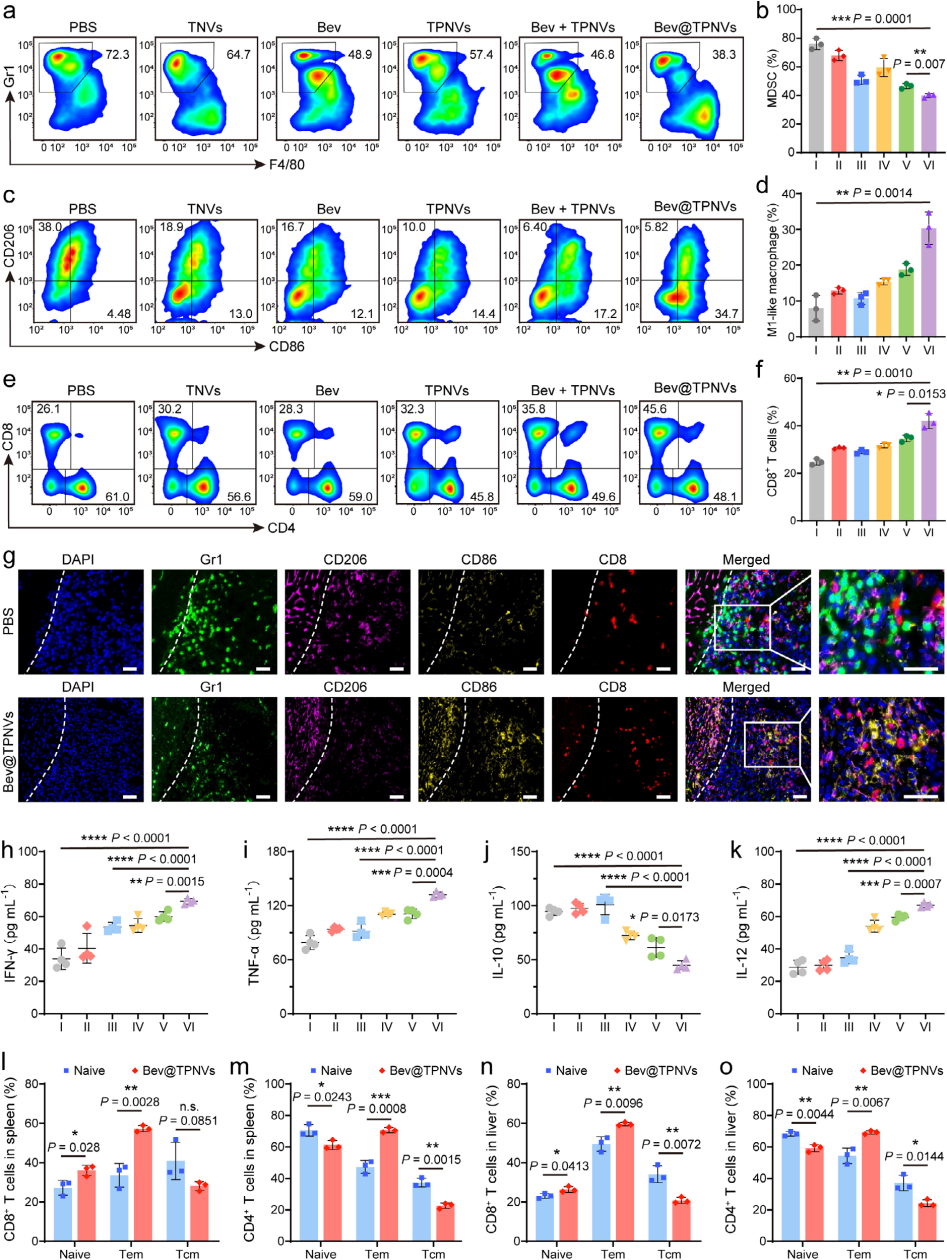
Bev@TPNVs trigger robust antitumor immune response and systemic immune memory effect. (a), (b) Representative flow cytometry (FCM) analysis (a) and quantification (b) of MDSCs (CD11b^+^Gr1^+^) in CD45^+^ cells (*n* = 3 biologically independent samples). (c), (d) Representative FCM analysis (c) of TAMs‐M1 (CD86^+^) and TAMs‐M2 (CD206^+^) in CD11b^+^F4/80^+^ cells and quantification of TAMs‐M1 (d) (*n* = 3 biologically independent samples). (e), (f) Representative FCM analysis (e) and quantification (f) of CD8^+^ T cells in CD45^+^CD3^+^ cells (*n* = 3 biologically independent samples). (g) Representative multiplex immunohistochemistry staining images of liver sections in the ablated transition zone (TZ) from PBS and Bev@TPNVs groups showing DAPI, Gr1^+^, CD206^+^, CD86^+,^ and CD8^+^ cells infiltration (Scale bars: 50 µm). The dotted lines are located at the outer edge of the necrosis. (h–k) Serum cytokine levels in serum obtained from mice after different treatments (*n* = 4 biologically independent samples). (l), (m) Flow cytometric quantification of effector memory T (Tem) cells and central memory T (Tcm) cells subset from CD8^+^ (l) and CD4^+^ (m) T cells in the spleens (*n* = 3 biologically independent samples). (n), (o) Flow cytometric quantification of Tem cells and Tcm cells subset from CD8^+^ (n) and CD4^+^ (o) T cells in the livers (*n* = 3 biologically independent samples). Groups: I: PBS, II: TNVs, III: Bev, IV: TPNVs, V: Bev + TPNVs, VI: Bev@TPNVs. All data are presented as mean ± SD. Statistical significance was calculated using a two‐tailed unpaired Student's *t*‐test. n.s., not significant, ^*^ *p* < 0.05, ^**^ *p* < 0.01, ^***^ *p* < 0.001, and ^****^ *p* < 0.0001.

To investigate the impact of Bev@TPNVs administration on liver tumor microenvironment, livers were collected on the 1st day post‐completion of various treatments (Figure 7a). FCM analysis revealed that treatment with Bev alone led to a reduction of MDSCs and facilitated the polarization of TAMs toward the M1 phenotype, and the combined administration of Bev and CD155/TIGIT blockade synergistically enhanced these effects by further decreasing MDSC levels and promoting TAMs‐M1 polarization (Figures 8a–d; S14). Furthermore, compared with the PBS group, although the CD4^+^ T cells in the Bev@TPNVs group decreased, the CD8^+^ T cells significantly increased (Figures 8e, f; S15). Notably, the ratios of CD8/MDSC and M1/M2, recognized as indicators of anti‐tumor immune balance, were the highest in the Bev@TPNVs group, aligning with its superior anti‐tumor efficacy observed in this group (Figure S16).

Subsequently, multiplex immunohistochemistry (mIHC) analysis was conducted to evaluate alterations in the immune microenvironment within the TZ and RZ. As anticipated, the Bev@TPNVs group displayed increased infiltration of TAMs‐M1 and CD8^+^ T cells in the TZ, coupled with reduced MDSCs and TAMs‐M2 infiltration (Figure 8g). Strikingly, the infiltration of these immune cells in the RZ was consistent with that in the TZ (Figure S17). At the same time, the spatial co‐localization of CD8^+^ T cells and CD155^+^ myeloid cells in the TZ and RZ significantly decreased after Bev@TPNVs treatment (Figure S18). The data demonstrated that Bev@TPNVs exerted their effects not only within the TZ but also effectively modulated the immune microenvironment throughout the entire liver. Additionally, the livers of the Bev@TPNVs treatment group demonstrated a significant reduction in Ki67 expression and mean vessel density (evaluated through CD31 staining), highlighting their potent ability to suppress tumor cell proliferation and neovascularization (Figure S19). Moreover, the augmented secretion of cytokines, including tumor necrosis factor‐α (TNF‐α), IFN‐γ, and interleukin‐12 (IL‐12), and decreased secretion of IL‐10 highlighted the capacity of Bev@TPNVs to stimulate innate and adaptive immune responses (Figure 8h–k).

Bev@TPNVs have been shown to activate local CD8^+^ T cells in the liver following the MWA of liver metastases, but the establishment of systemic immune memory remained unconfirmed. Memory T lymphocytes consist of effector memory T (Tem) cells and central memory T (Tcm) cells [32]. Tem cells are capable of migrating to inflamed peripheral tissues and providing immediate protective functions [32, 33]. In our study, livers and spleens of mice were collected 18 days post‐treatment, with sex‐ and age‐matched naive mice serving as controls. Notably, compared to naive mice, there was a significant shift of Tcm (CD62L^+^CD44^+^) in CD8^+^ T cells and CD4^+^ T cells toward Tem (CD62L^−^CD44^+^) phenotype in thespleens and livers of long‐term surviving mice following Bev@TPNVs treatment (Figures 8l–o; S20 and S21).

## 3 Conclusions

The recurrence of liver metastases following ablation remains a significant challenge in thermal ablation therapy, and currently, there is no efficacious treatment available. Previous studies have demonstrated that the higher rates of tumor recurrence observed after ablation can be partially attributed to the challenge of achieving perioperative margins exceeding 5 mm, leading to residual tumor mass. However, even thermal ablation performed on normal liver tissue to obtain a sufficient incisal margin may result in the accumulation of micro‐metastases within the ablated TZ [12]. Therefore, irrespective of the etiology of local recurrence, there is an urgent need to gain insights into how the post‐ablation microenvironment exerts an aggressive phenotype on tumor cells in the TZ.

Here, based on a preclinical CRLM model, we observed that the ablation of liver metastases stimulated tumor proliferation in the ablated TZ, resulting in shortened survival time in mice. Subsequently, we explored the alterations in cell populations and immunophenotypes in the TZ post‐MWA of liver metastases at the single‐cell level. Our data showed that VEGF‐mediated immunosuppression was accompanied by significant expression of CD155 in myeloid cells, rendering them responsive to immunotherapy targeting tumor angiogenesis and the CD155/TIGIT pathway. Our study not only revealed the previously unrecognized characteristics of the immune microenvironment in the ablated TZ of liver metastases, but also proposed an innovative therapeutic target for combined therapy following liver metastasis ablation. Besides, these findings may serve as a valuable resource for investigating the biological insights after ablation of liver metastases.

Previous studies have investigated adjunctive treatment strategies for thermal ablation of liver metastases. In a phase II clinical trial, patients with liver metastases who underwent thermal ablation received systemic chemotherapy as adjuvant treatment; however, no significant improvement in recurrence‐free survival (RFS) was observed compared to thermal ablation alone [34]. Similarly, thermal ablation‐assisted two‐step hepatectomy (liver partitioning and portal vein occlusion for staged hepatectomy, ALPPS) also failed to enhance RFS [35]. Currently, there is no effective adjuvant therapy following the thermal ablation of liver metastases, highlighting the urgent need for new therapeutic strategies. Modulating the immune microenvironment to bolster anti‐tumor immunity may represent a viable strategy to prevent recurrence subsequent to liver metastasis ablation. Preclinical studies have indicated that anti‐programmed death‐1 (PD‐1) therapy can enhance the anti‐tumor immune response associated with thermal ablation [36], but the limited efficacy is due to the low infiltration of T cells in the microenvironment. Further clinical and preclinical studies are needed. By comparison, Bev@TPNVs possesses three core advantages, providing an innovative solution for the intervention of liver metastasis recurrence after ablation. First, the platelet membrane fragments carried by Bev@TPNVs retain the natural homing property of platelets to damaged blood vessels, enabling specific recognition of the damaged blood vessels in the TZ area induced by MWA and achieving efficient enrichment of the carrier in the TZ. This characteristic not only significantly improves the bioavailability of Bev and TIGIT in the target area but also significantly reduces off‐target toxicity and enhances the biological safety of the treatment. Second, different from single‐target intervention strategies, Bev@TPNVs can simultaneously intervene in VEGF‐mediated angiogenesis and CD155/TIGIT‐mediated immune suppression, while solving the two key problems of tumor growth‐dependent “nutrient supply” and “immune evasion”, forming a synergistic therapeutic effect. Finally, it can induce systemic long‐term immune memory, promoting the transformation of Tcm in the liver and spleen into Tem, which can remain in the peripheral tissues for a long time and quickly initiate anti‐tumor immune responses when the tumor reappears, providing continuous anti‐tumor protection and effectively reducing the long‐term recurrence risk.

In addition, we explored the optimal time window for immunotherapy after ablation of liver metastases. In mouse models of CRLM, focal MWA to the liver ultimately promotes tumor progression in the ablated TZ, so understanding the dynamics of these tumorigenesis processes is crucial for developing effective combination treatment strategies to counter this phenomenon. We observed significant increases in tumor burden, angiogenesis, and tumor proliferation in the TZ starting from day 3 post‐ablation, suggesting that the time window for benefit from combination therapy is within 3 days. The results showed that initiating treatment 1 day post‐MWA significantly inhibited the progression of liver metastases in mice.

Finally, the Bev@TPNVs we have prepared are expected to serve as a comprehensive treatment regimen that can effectively complement local therapies for liver metastases, encompassing not only ablation but also radiotherapy and surgery. Preclinical and clinical studies suggest that radiotherapy and surgery can promote local tumor proliferation and neovascularization, inducing immunosuppressive effects of myeloid infiltration, which are considered a crucial mechanism for tumor recurrence [37]. Our data suggested that the proposed combination therapy notably hindered neovascularization and improved the liver microenvironment. Specifically, the proliferation of MDSCs in the liver was significantly inhibited, TAMs were polarized toward the M1 phenotype, and the anti‐tumor activity of CD8^+^ T cells was significantly activated. Surprisingly, these changes occurred not only in the ablated TZ but also in the RZ at the distal end of ablation, suggesting a systemic improvement in liver biology.

In summary, we delved into the consequences of thermal ablation on disseminated liver metastases and identified the optimal time window for post‐ablation immunotherapy. Our proposed combined immunotherapy strategy not only provides unique insights into the immunobiology of liver metastases post‐ablation, but also opens avenues for targeting therapeutic pathways in liver metastases ablation therapy. The successful clinical translation of this strategy holds the promise of revolutionizing the treatment landscape for liver metastasis patients, offering a beacon of hope to a broader patient population.

## Funding

This work was supported by the National Natural Science Foundation of China to S.L.P (Grant No. 82171945) and X.H.X (Grant No. 82151318), Shanghai Municipal Health Commission grant to X.H.X (Grant No. SHSLCZDZK03502), Natural Science Foundation of Heilongjiang Province to L.S.Y (Grant No. JJ2025QC0429), China Postdoctoral Science Foundation to L.S.Y (Grant No. 2025MD784136), and Shanghai Anti‐Cancer Association to S.Y.T (Grant No. SACA‐CY25B06).

## Conflicts of Interest

The authors declare no conflicts of interest.

## Supporting information




**Supporting File 1**: advs74270‐sup‐0001‐SuppMat.pdf.

**Supporting File 2**: advs74270‐sup‐0002‐Data.zip.

## Data Availability

The scRNA‐seq data generated in this study have been deposited in the Genome Sequence Archive (GSA) database under accession code CRA025945. The remaining relevant data that support the findings of this study are available from the corresponding author upon reasonable request.
