# Supplementary material for: Engineered TIGIT‐Blockade Membrane Vesicles Synergize with Microwave Ablation to Mediate Liver Metastases Eradication
Source: Adv Sci (Weinh). 2026 Feb 8;13(22):e22918. doi: 10.1002/advs.202522918 (PMC13088283; doi:10.1002/advs.202522918)
Supplement: Supplementary file 1 — Supporting File 1: advs74270‐sup‐0001‐SuppMat.pdf. [file ADVS-13-e22918-s002.pdf]

Supporting Information

Engineered TIGIT-Blockade Membrane Vesicles Synergize with Microwave Ablation to Mediate Liver Metastases Eradication

Shaoyue Li, Weichen Xu, Yuting Shen, Xuexia Shan, Jiawei Sun, Shisi Ding, Xiaodong Hou, Shaoning Zhang, Zhiyuan Niu, Taixia Wang, Xin Guan, Xiao Li, Weiwei Ren, Dou Du, Huixiong Xu, Wenwen Yue*, and Liping Sun**

Experimental Section

Materials: All chemicals in our study were obtained from Sigma-Aldrich unless otherwise specified. Dipalmitoyl phosphatidylcholine (DPPC), 1,2-distearoyl-sn-glycero-3phosphoethanolamine-N-[amino(polyethylene glycol)2000] (DSPE-PEG-2000), 1,2-distearoyl-sn-glycero-3phosphocholine (DSPC), and cholesterol were obtained from Xi'an Ruixi Biological Technology Co., Ltd. Mouse IL-10, IL-12, IFN-γ and TNF-α ELISA Kits were purchased from YOBIBIO (Shanghai, China). Bevacizumab was purchased from MedChemExpress (Catalog No. HY-P9906). The anti-CD61 antibody (Catalog No. ab179473) and anti-TIGIT antibody (Catalog No. ab300073) were purchased from Abcam.

Cell lines and mice: CT26 murine colorectal cancer-cell line (Catalog No. TCM37) was obtained from Cell Bank/Stem Cell Bank, Chinese Academy of Sciences. fLuc-CT26 cells (Catalog No. LZQ0032) and HEK-293T cell line (Catalog No. ZQ0358) were purchased from Shanghai Zhong Qiao Xin Zhou Biotechnology Co. Ltd. All cell lines used in this study were quarterly authenticated and ensured to be free of mycoplasma contamination. Cells were maintained in the recommended medium and conditions. All mouse experiments were conducted in accordance with the policies of the National Ministry of Health and the protocols approved by the Laboratory Animal Center of Shanghai Tenth People's Hospital. The assigned approval/accreditation number is SHDSYY-2022-1600266-2. Female BALB/c mice aged 6-8 weeks were purchased from Jiangsu Huachuang Sino Pharma Tech Co., Ltd.

Establishment of ablation model of colorectal liver metastases: Through a left lateral flank incision, 5 × 10^5 CT26 cells or Luc-CT26 cells were injected into the splenic parenchyma. After 10 min, half of the spleen receiving the tumor cells was removed to prevent intrasplenic tumor growth. Diffuse intrahepatic metastases were allowed for 3 days. Three days after the intrasplenic injection, a single MWA treatment was initiated using a microwave generator (ECO-100E, Yigao Microwave Electric Institute, Nanjing, China). After anesthesia, the mice were exposed to the liver under sterile conditions, and the MWA antenna was inserted percutaneously into the left lobe of the liver under sterile conditions. Treatments were controlled for 1.0 min with the ablation power set at 5 Watts.

Tumor analysis: The tumor burden in the liver was determined by the hepatic replacement area (HRA) score, namely the percentage of liver tissue was replaced by the tumor tissue. HRA was measured in transition zone (TZ) (defined as the area 2 mm outside the necrotic center area) and reference zone (RZ) (i.e., the remaining part of the liver). H&E images were captured under at 4× magnification and quantitative analysis was performed using the QuPath software (version 0.6.0).

Single-cell RNA sequencing: Under sterile conditions, freshly harvested TZ liver tissues on day 3 post-MWA and micrometastasis control liver tissues were processed into single-cell suspensions. The concentration of the resulting single-cell suspensions was adjusted to 700-1200 cells μL^{-1}. Library construction and loading were performed in accordance with the manufacturer's instructions for the 10 × Genomics Chromium Next GEM Single Cell 3' Reagent Kits v3.1 (Catalog No. PN-1000268). The constructed libraries were subjected to high-throughput sequencing on the Illumina NovaSeq 6000 platform with a paired-end read length of 150 base pairs (PE150).

Isolation of $CD155^+$ myeloid cells, $CD155^+$ pDCs and $CD8^+$ T Cells: The ablated TZ tissues were harvested to obtain single-cell pellets. For isolation of $CD155^+$ myeloid cells ($CD11b^+CD155^+$) and $CD155^+$ pDCs ($Siglech^+CD155^+$), the single-cell suspension was first blocked with anti-mouse CD16/32 antibody (BioLegend, Catalog No. 156614) to prevent non-specific antibody binding. Then, the cells were stained with fluorescent dye-conjugated antibodies, including CD11b-BV711 (BD Biosciences, Catalog No. 563168), CD155-PE-Cy7

(BioLegend, Catalog No. 131512), and Siglech-FITC (BioLegend, Catalog No. 129604). $CD155^+$ myeloid cells and $CD155^+$ pDCs were isolated using a BD FACSAria III FACS system. For $CD8^+$ T cell purification, mouse splenic single-cell suspensions were processed using magnetic-activated cell separation (MACS) with anti-CD8 magnetic beads (STEMCELL, Catalog No. 17853). The extracted $CD8^+$ T cells were then stimulated with recombinant inter-leukin-2 (IL-2; R&D Systems, USA) and CD3/CD28 T Cell Activator (STEMCELL, 10971).

$CD8^+$ T cell co-culture suppression assay: Purified $CD8^+$ T cells were activated with 5 μg mL^{-1} concanavalin A (ConA, Sigma). The activated $CD8^+$ T cells were then co-cultured with isolated $CD155^+$ myeloid cells or $CD155^+$ pDCs at a cell ratio of 10:1 in 24-well plates. For pathway blockade experiments, anti-CD155 antibody (Thermo Fisher Scientific, Catalog No. MA5-29762) was added to the co-culture system at a final concentration of 10 μg mL^{-1}. The co-culture system was incubated at 37 °C with 5% CO_2 for 3 days. After co-culture, the cells were harvested and stained for FCM analysis. The cells were first blocked with anti-CD16/32 antibody, then stained with CD3-BV421 (BD Biosciences, Catalog No. 562600), CD8-PE-Cy7 (eBioscience, Catalog No. MHCD0812), and CD69-FITC (BioLegend, Catalog No. 104506) antibodies. For intracellular IFN-γ detection, the cells were fixed and permeabilized using a Fixation/Permeabilization Kit (eBioscience) according to the manufacturer's protocol, followed by staining with IFN-γ-PE (BioLegend, Catalog No. 505808) antibody. The stained cells were analyzed using a BD LSRFortessa X-20 flow cytometer, and the data were processed with FlowJo software.

Phenotypic analysis of $CD155^+$ myeloid cell subsets: Single-cell suspensions from TZ tissues of control and MWA groups were prepared as described above. The cells were blocked with anti-CD16/32 antibody, then stained with CD45-BV510 (BioLegend, Catalog No. 103137), CD11b-BV711, Gr1-PerCP-Cy5.5 (BioLegend, Catalog No. 108427), F4/80-BUV396 (BD Biosciences, Catalog No. 750644), and CD155-PE-Cy7 antibodies. For TAM phenotypic analysis, additional antibodies including CD86-APC (Biolegend, Catalog No. 159216) and CD206-PE (Biolegend, Catalog No. 141705) were used for staining. All stained cells were analyzed by FCM, and all data were quantified using FlowJo software.

VEGF signaling inhibition assay: The mouse model of colorectal liver metastases (CRLM) was established using the method described above. At 6 h post-MWA, mice were intravenously injected via the tail vein with either anti-VEGF antibody (Bev) or isotype-matched control antibody (MedChemExpress, Catalog No. HY-P99001) at a dose of 5 mg kg^{-1} daily for a total of three consecutive administrations. Three days post-MWA, all mice were euthanized. Liver tissues from the TZ of experimental mice and liver tissues with micrometastases from control mice were harvested and processed into single-cell suspensions. The resulting cell suspensions were pre-blocked with an anti-CD16/32 antibody, followed by staining with CD45-BV510, CD11b-BV711, and CD155-APC antibodies. Stained cells were subsequently analyzed by FCM.

Preparation and characterization of TIGIT engineered cells: HEK-293T cells were transfected with a vector carrying purinamycin resistance gene. To construct TIGIT engineered cells, HEK-293T cells were infected with a lentivirus that fused the mouse TIGIT gene with the EGFP tag. The cells were cultured in 5% CO_2 and maintained in Dulbecco's modified Eagle's medium (DMEM) supplemented with 10% fetal bovine serum (FBS) and 2 μg mL^{-1} purinamycin. After two weeks, cells that stably expressed TIGIT-EGFP were screened and continued to be maintained in complete medium containing 2 μg mL^{-1} purinomycin. The TIGIT-EGFP cells were detected by FCM and confocal laser scanning microscopy (CLSM) (Carl Zeiss, LSM 900). In addition, HEK-293T cells were infected with a lentivirus encoding the mouse TIGIT gene without the EGFP tag, and TIGIT cells were established.

Synthesis and characterization of TPNVs: First, TNVs and PNVs were prepared. For TNVs, TIGIT-EGFP cells were first collected by low-velocity centrifugation (500 × g, 5 min). TIGIT-EGFP cells were lysed in hypotonic lysis buffer for 5-10 min and destroyed by Dounce homogenizer. The solution was centrifuged at 3000 × g for 15 min to collect the supernatant. The supernatant was further centrifuged at 20000 × g for 30 min to obtain TNVs. Then the obtained TNVs were purified by washing 3 times with PBS containing protease inhibitors. Ultrasound was carried out for 5 min, and finally extruded by a mini extruder (Avanti Polar Lipids) through porous membrane filters of 1-, 0.4-, 0.2- and 0.1- μm polycarbonate.

For PNVs, 10 mL whole blood of healthy BALB/c mice was collected and centrifuged at 100 × g for 15 min. The supernatant was centrifuged at 800 × g for 20 min. The obtained precipitates were washed three times with PBS, frozen at -80 °C, thawed at room temperature, and centrifuged at 5000 × g for 3 min to obtain PNVs.

To prepare TPNVs, the protein concentrations of TNVs and PNVs were determined, and then TNVs and PNVs were mixed with a 4:1 protein mass ratio. Sonicated for 5 min, and then extruded using the mini extruder through a 0.1 μm polycarbonate porous membrane filter.

Droplets containing NVs were exposed to copper mesh for 60 s, then negatively stained with uranyl acetate for 30 s, and the morphology of multiple NVs was observed by transmission electron microscopy (TEM) (FEI Tecnai G2 F20, accelerating voltage = 200 kV). The potential and particle size of multiple NVs were detected by dynamic light scattering (DLS) (ZSU3100, Malvern Instruments, UK). In order to verify the fusion of different types of NVs, TNVs and PNVs were labeled with 3,3'-dioctadecyloxacarbocyanine perchlorate (DiO) (Beyotime Biotechnology, Catalog No. C1038) and 1,1'-dioctadecyl-3,3,3',3'-tetramethylindodicarbocyanine, 4-chlorobenzenesulfonate salt (DiD) (Beyotime Biotechnology, Catalog No. C1039), respectively, and then observed under CLSM after fusion.

Synthesis of liposomes: DPPC, DSPE-PEG-2000, and cholesterol (at a mass ratio of 5:1:1, totaling 10 mg) were dissolved in chloroform. The solvent was then evaporated using a rotary evaporator under conditions of 35 °C 100 rpm, and 50 mbar to form a lipid film. The lipid film was hydrated with PBS and stirred at 37 °C for 1 h to assemble liposomes. Following 5 min of ultrasonic treatment, the liposome suspension was sequentially filtered through a mini-extruder equipped with polycarbonate membrane filters of decreasing pore sizes (1 μm, 0.4 μm, 0.2 μm, and 0.1 μm).

Western blotting: Total protein concentration of multiple NVs was assessed using a BCA protein assay kit. An equal amount of protein (20 μg) was separated in 8-12% sodium dodecyl sulfate polyacrylamide gel. The protein was transferred to PVDF membrane and incubated with blocking buffer at 25 °C for 1 hour. The membrane was incubated overnight with anti-CD61 antibody and anti-TIGIT antibody (1:1000 dilution), respectively, followed by immersion in the coupled secondary antibody at room temperature for 1 hour. After three

washes, protein expression levels of multiple NVs were measured by chemiluminescent imaging system (GE Healthcare, USA).

Measurement of the drug loading efficiency (DLE) of Bev: Bev was incorporated into TPNVs via three distinct methods: incubation at room temperature (RT), freeze-thaw cycling (FT), and sonication treatment. For the freeze-thaw cycling method, 50 μg of Bev was introduced into 250 μg of TPNVs (dissolved in 1 mL of PBS). The resulting mixture was first frozen at -80 °C for 30 min and then incubated at room temperature for 30 min. This freeze-thaw process was repeated three times. For the sonication treatment method, the mixture underwent sonication under the conditions of 500 V, 2 kHz frequency, 20% power, with 3 cycles, each cycle consisting of 6-second pulses and 3-second pauses. After sonication, it was cooled on ice for 30 min. Regarding the determination of DLE, the amount of Bev encapsulated within TPNVs was calculated by taking the difference between the total amount of Bev used in the TPNVs formulation and the quantity of Bev present in the supernatant after ultra-centrifugation at 40000 x g for 30 min. The free Bev in the supernatant was quantitatively analyzed by high performance liquid chromatography (HPLC, LC-10Atvp, Shimadzu, Kyoto, Japan). The DLE was calculated taking into account the total dry weight of TPNVs and determined as follows:

$$\text{DLE (\%)} = \frac{\text{total amount of Bev} - \text{free Bev in supernatant}}{\text{total dry weight of TPNVs}} \times 100$$

Bev In vitro drug release study: To assess the release amount of Bev from TPNVs, the mixture of Bev and TPNVs, post-sonication, was initially subjected to ultra-centrifugation at a force of 40000 x g for 30 min. This step effectively removed any unencapsulated Bev. Subsequently, the Bev-loaded TPNVs were dispersed in 20 mL of PBS buffer and incubated with magnetic stirring at 37 °C for 30 min. At pre-determined time intervals (0, 1, 2, 4, 8, 12, 24, 48, 72 h), the samples were ultra-centrifuged under the identical conditions as described above. After centrifugation, 200 μL of the supernatant was collected. The samples were then supplemented with an equal volume of fresh PBS. Finally, the release amount of Bev in the collected supernatant was calculated using HPLC.

In vitro biological effects and in vivo perinecrotic targeting: The effect of drug-loaded

cell membrane vesicles on the activity of CT26 cells was detected via standard CCK-8 assay. In order to evaluate the targeting effect of TPNVs, a post-ablation model of CRLM was first established according to the above protocol. 100 μg CY5.5-NHS-labeled liposomes, TNVs, PNVs, and TPNVs were injected through the tail vein of mice one day after ablation. 25 μL of peripheral blood were harvested at 2 h, 4 h, 8 h, 16 h, 24 h, and 48 h after injection and diluted with 25 μL of PBS. Fluorescence imaging of the diluted blood samples was performed via *in vivo* imaging system (IVIS) spectrum imaging system (VISQUE Invivo Smart-LF). The isolated heart, liver, lung and kidney were taken for organ fluorescence imaging at 48 h after injection.

Treatment and monitoring experiments after MWA of liver metastases: In a Luc-CT26 liver metastasis model, mice underwent MWA on the third day followed by treatment with PBS, TNVs, Bev, TPNVs, Bev + TPNVs, or Bev@TPNVs (25 mg of TNVs or TPNVs and 5 mg of Bev per kilogram body weight) on days 4, 7, 10, and 13 (n = 7 mice per group). Bioluminescence signals from Luc-CT26 cells were taken via the IVIS imaging system to assess liver metastasis progression. On day 18, metastatic burden was assessed through H&E staining of liver tissue sections and liver weight measurements to evaluate the therapeutic efficacy of Bev@TPNVs. In the CT26 liver metastasis model, mice underwent MWA on the third day and the followed therapy strategy of treatment group was the same as above. Mice were weighed and arranged to evaluate survival curves (n = 10 mice per group). On the 1st day after the completion of various treatments, the liver samples were collected for immunology analysis (n = 3 mice per group).

Flow cytometry assay: Single cell suspension (10^6 cells in total volume 100 μL) was obtained from isolated mouse livers. The harvested cells were then pre-incubated with anti-CD16/32 monoclonal antibodies for 15 min at 4 °C and stained with the following fluorescent dye conjugated antibodies diluted 100 times: CD45-BV510, CD3-APC (eBioscience, Catalog No. 17-0032-82), CD4-BV786 (BD Biosciences, Catalog No. 568368), CD8-PE-Cy7, CD11b-BV711, F4/80-BUV396, CD86-BV421 (Biolegend, Catalog No. 105123), CD206-PE, and Gr1-PerCP-Cy5.5 were then analyzed by FCM. To analyze memory T cells, Live-NIR, CD3-PE (eBioscience, Catalog No. 25-0031), CD4-BV786, CD8-PE-Cy7, CD44-FITC

(eBioscience, Catalog No. MHCD4401) and CD62L-APC (eBioscience, Catalog No. A14720) were stained and analyzed with FCM. All data were analyzed using FCM analysis software.

Multiplex immunohistochemistry (mIHC) assay: mIHC was performed by using 5/4-Color Multiple IHC Kit (Absin Bioscience Inc., Shanghai, China). Various treated liver tissues were collected from mice. Frozen microtome was used to cut liver sections containing ablation areas, pasted on slides, and fixed with different primary antibodies: CD11b (Cell Signaling Technology, Catalog No. 93169), CD155 (Thermo Fisher Scientific, Catalog No. MA5-29762), Gr1 (Proteintech, Catalog No. 65140-1), CD206 (Cell Signaling Technology, Catalog No. 24595), CD86 (Cell Signaling Technology, Catalog No. 19589), and CD8 (Abcam, Catalog No. 217344). Then added the corresponding secondary antibodies following the manufacturer's instructions. Nuclei was counterstained for DAPI. The obtained images were analyzed using Halo software (V.3.1).

Cytokine analysis: The serum was collected from mice after different treatments. Enzyme-linked immunosorbent assay (ELISA) kits were used to measure the serum levels of tumor necrosis factor-α (TNF-α) (YOBIBIO, Catalog No. U96-3112E), interferon-γ (IFN-γ) (YOBIBIO, Catalog No. U96-1475E), interleukin (IL)-10 (YOBIBIO, Catalog No. U96-1517E) and IL-12 (YOBIBIO, Catalog No. U96-1522E) according to the manufacturer's instructions.

Immunofluorescence staining: Livers were extracted from mice and quickly frozen at optimal cutting temperatures. The liver sections were cut with a cryotome and placed on a slide. Sections were stained with various primary antibodies: anti-Ki67 antibody (Cell Signaling Technology, Catalog No. 9449) and anti-CD31 antibody (Cell Signaling Technology, Catalog No. 3528), as per manufacturer's instructions. Confocal microscopy (Pannoramic MIDI, 3Dhistech) was used to analyze the slides.

Statistical analysis: Data were shown as means ± standard deviation (SD), as indicated. Statistical differences were calculated through GraphPad Prism software (version 9.0.0) using using two-tailed unpaired student's *t*-test, and denoted as n.s., not significant, $^{*}P < 0.05$, $^{**}P < 0.01$, $^{***}P < 0.001$, and $^{****}P < 0.0001$. All flow cytometry data were analyzed on FlowJo™ software package (version 10.5.2). Quantitative analyses of H&E and fluorescence

images were performed using image J software (version 1.8.0). Bioluminescent and fluorescent images were analyzed on CleVue (version 3.1.3.2054).

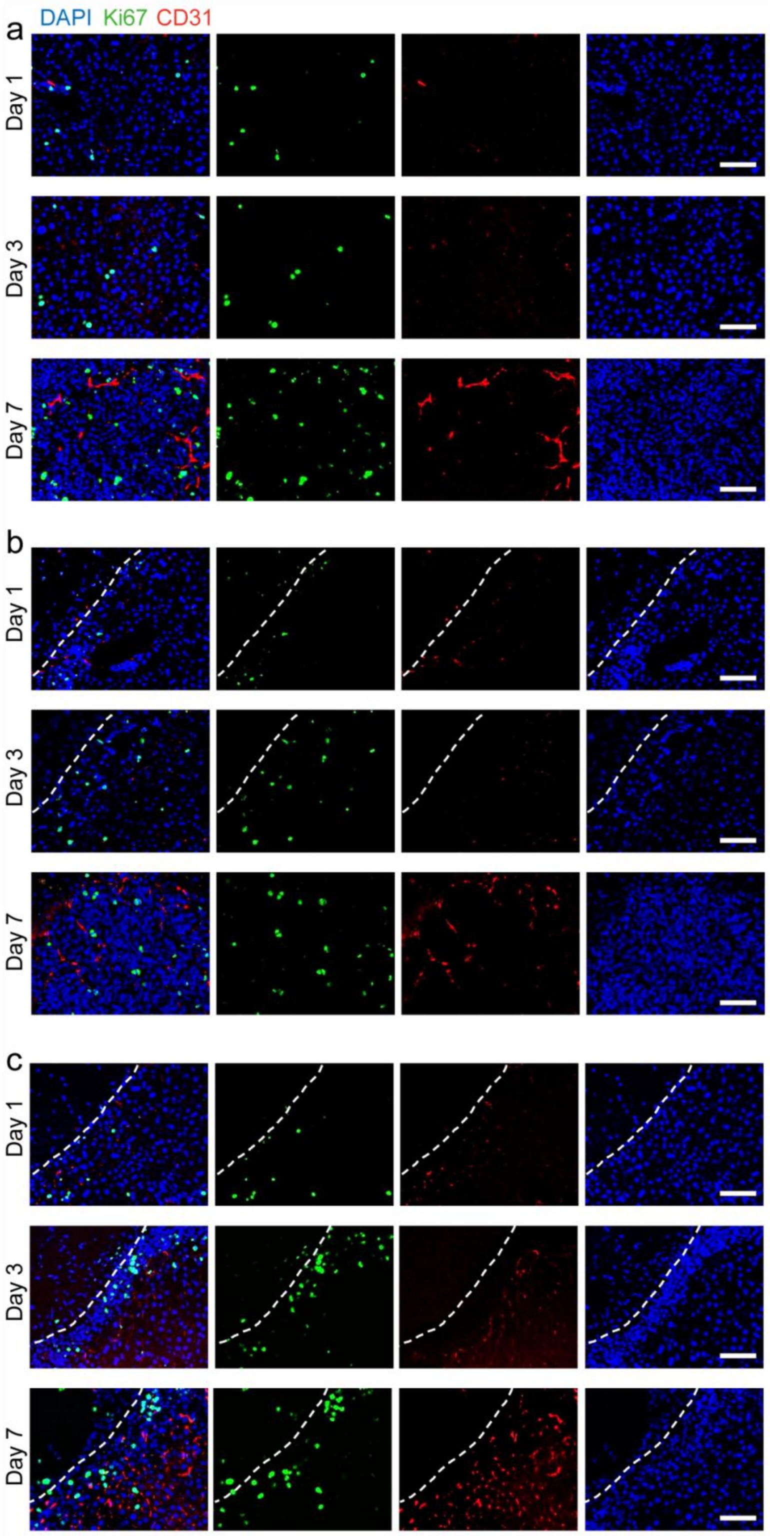

Figure S1. (a-c) Representative immunofluorescence images at different time points displaying the infiltration of Ki67 and CD31 in the reference zone (RZ) of the control group (a) and the sham-operated group (b), as well as in the ablated transition zone (TZ) of the microwave ablation (MWA) group (c) (Scale bars: 50 μm). The dotted lines are located at the outer edge of the injury or necrosis.

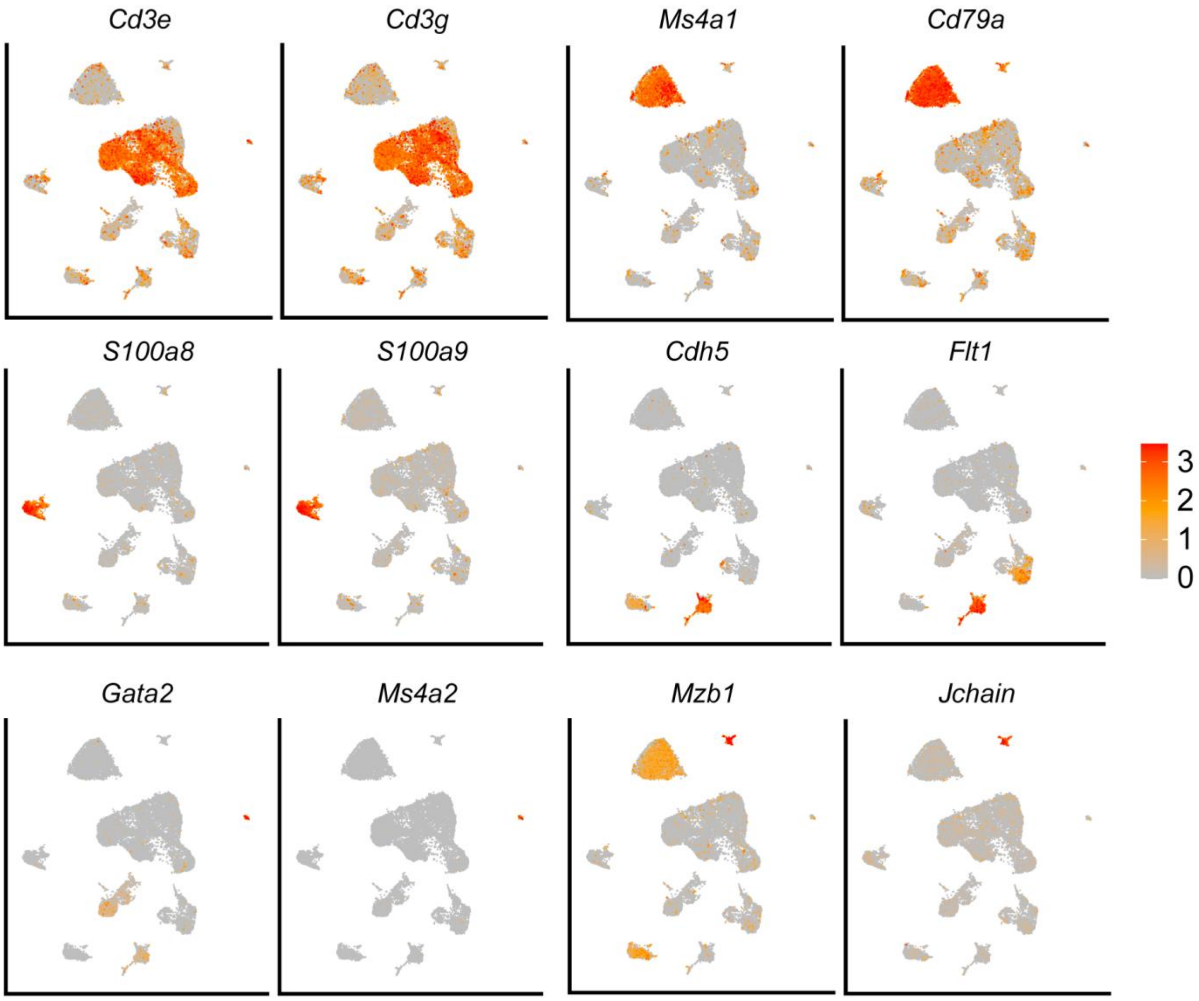

Figure S2. Expression levels of selected classical marker genes in unsorted cells shown in UMAP plots from both control and transition zone (TZ) tissues in liver metastasis mice.

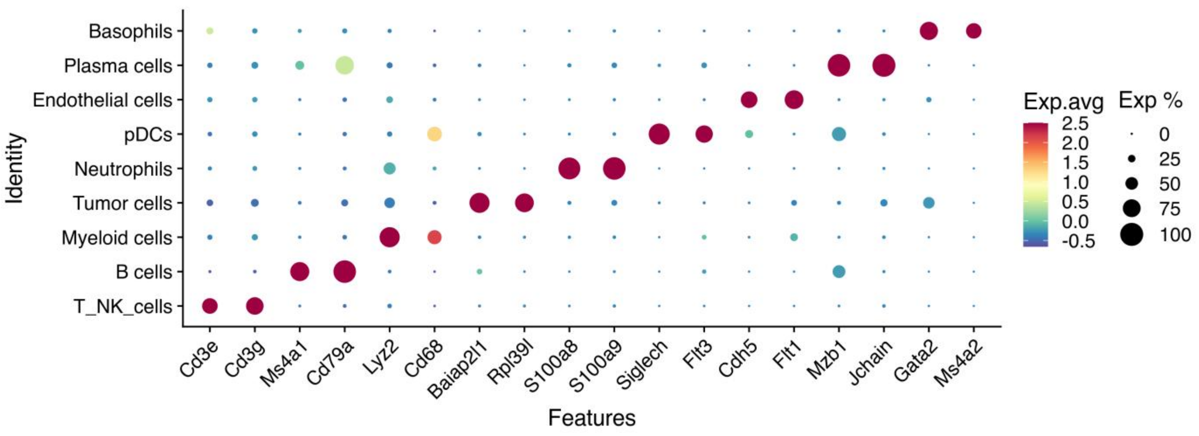

Figure S3. Dot plots showing average expression of known markers in indicated cell clusters. The dot size represents percent of cells expressing the genes in each cluster.

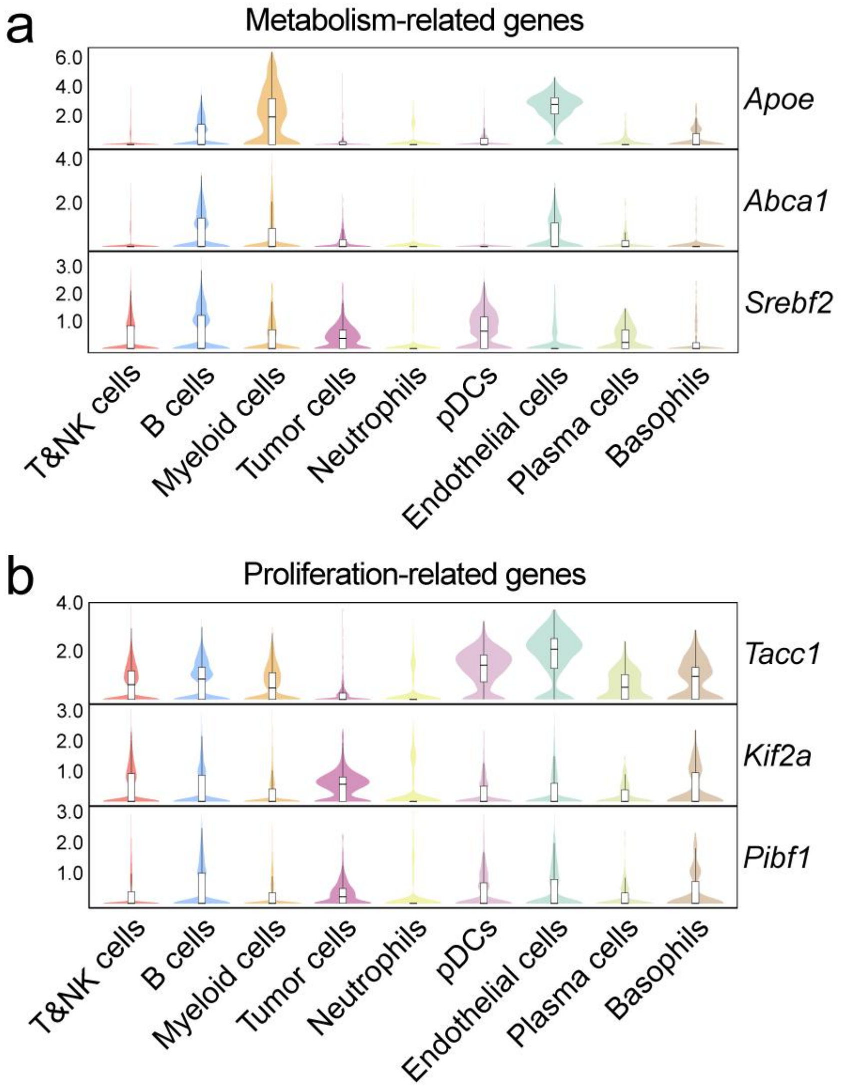

Figure S4. Violin plots showing the probability distribution of gene expression related to metabolism and proliferation in different cell clusters.

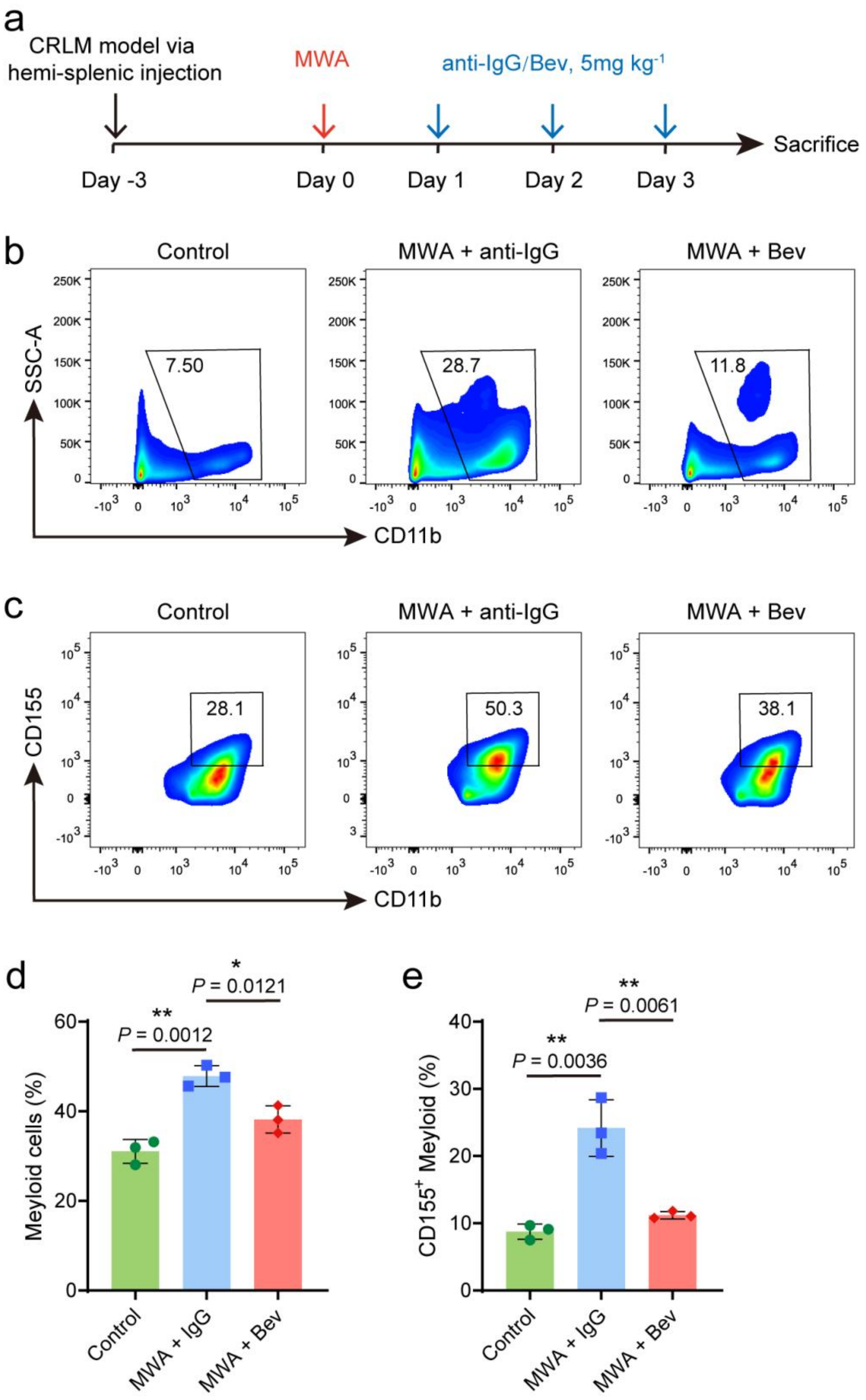

Figure S5. (a) Schematic diagram of the experimental design. **(b, c)** Representative flow cytometry (FCM) analysis of myeloid cells ($CD11b^+$) (b) and $CD155^+$ myeloid cells ($CD11b^+CD155^+$) (c) in $CD45^+$ cells (n = 3 biologically independent samples). **(d, e)** Corresponding quantification of myeloid cells (d) and $CD155^+$ myeloid cells (e) (n = 3 biologically independent samples). All data are presented as mean ± SD. Statistical significance was calculated using two-tailed unpaired Student's *t*-test. **P* < 0.05 and ***P* < 0.01.

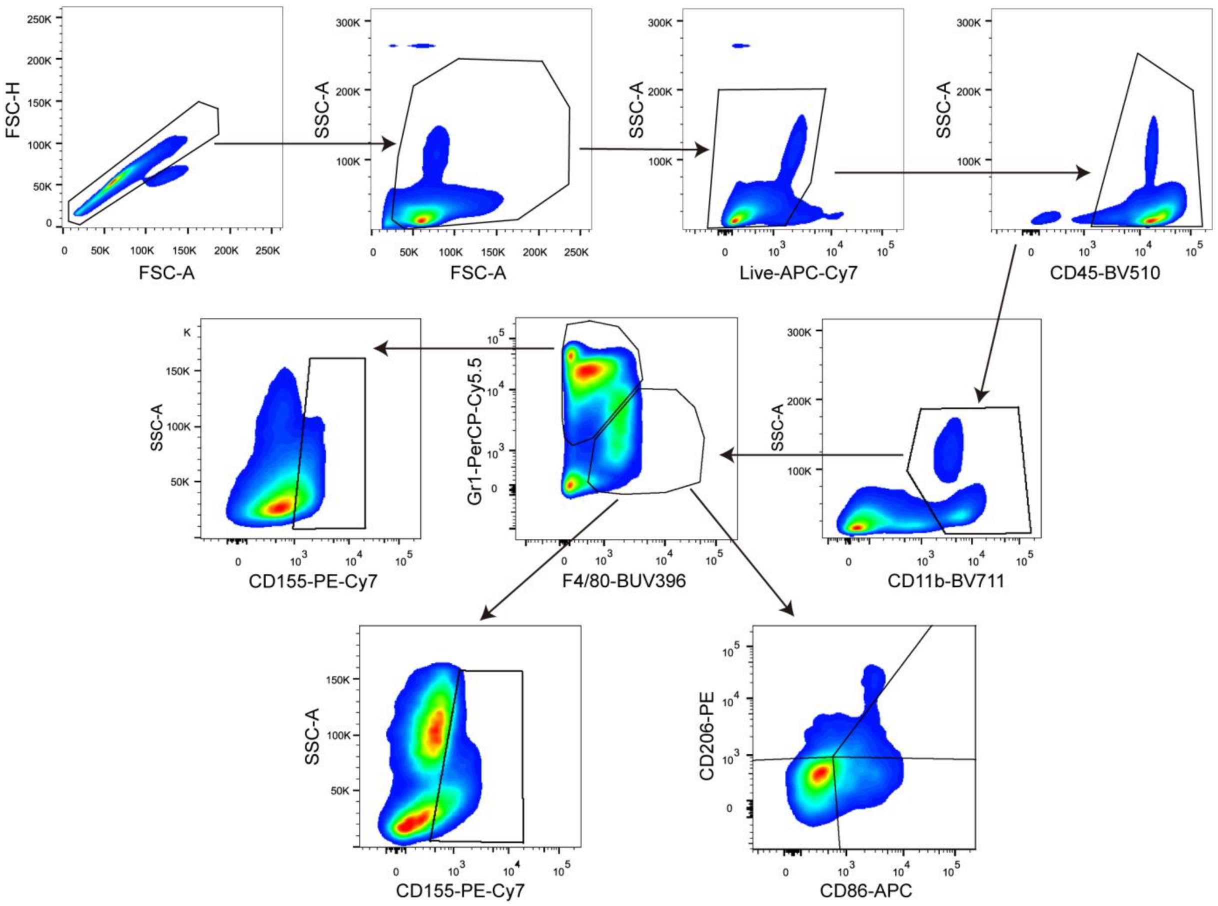

Figure S6. Gating scheme for analyzing myeloid-derived suppressor cells (MDSCs), tumor-associated macrophages (TAMs), CD155$^+$ MDSCs, CD155$^+$ TAMs, TAMs-M1, and TAMs-M2 in the tissue of the control and ablated transition zone (TZ) of liver metastases using flow cytometry.

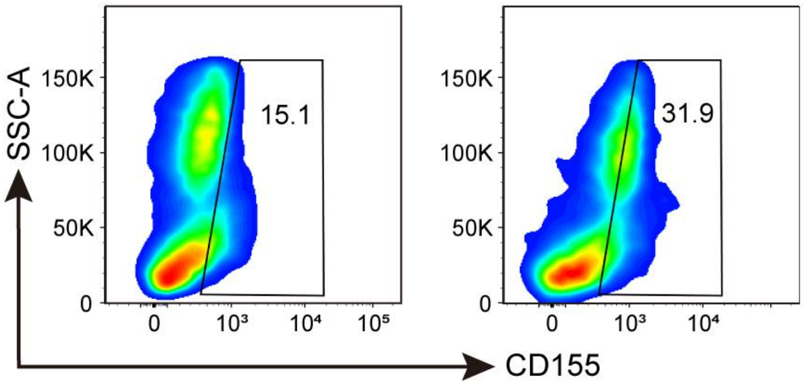

Figure S7. Representative flow cytometry (FCM) analysis of CD155$^+$ TAMs (CD11b$^+$F4/80$^+$CD155$^+$) in CD45$^+$ cells.

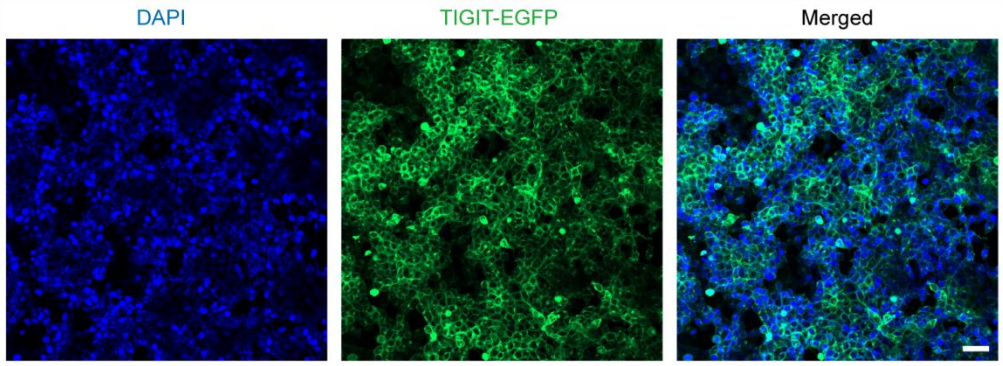

Figure S8. Confocal images of TIGIT protein expression on the engineered human embryonic kidney (HEK)-293T stable cell lines. (Scale bar: 50 μm).

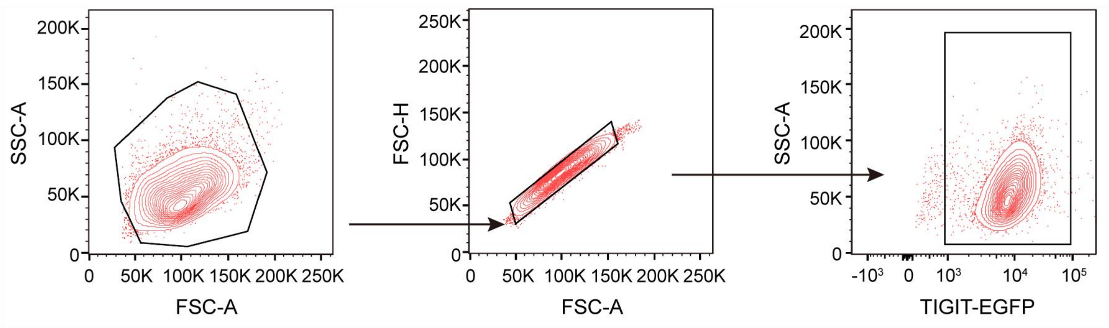

Figure S9. Gating scheme for analyzing TIGIT protein expression on the engineered HEK-293T cells using flow cytometry.

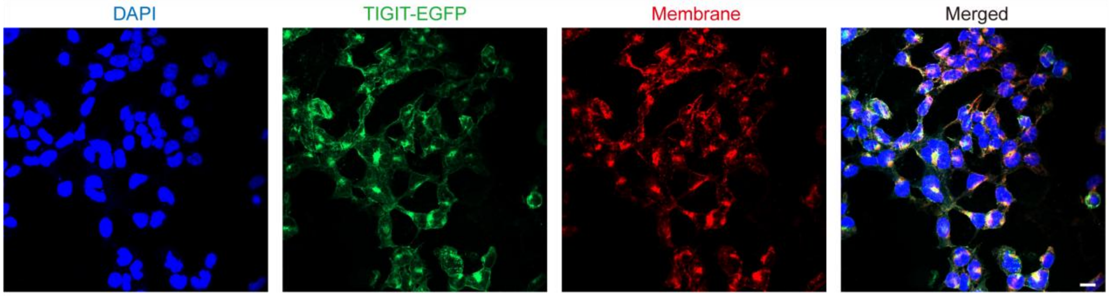

Figure S10. Confocal images of TIGIT protein expression on the cell membrane (Scale bar: 10 μm).

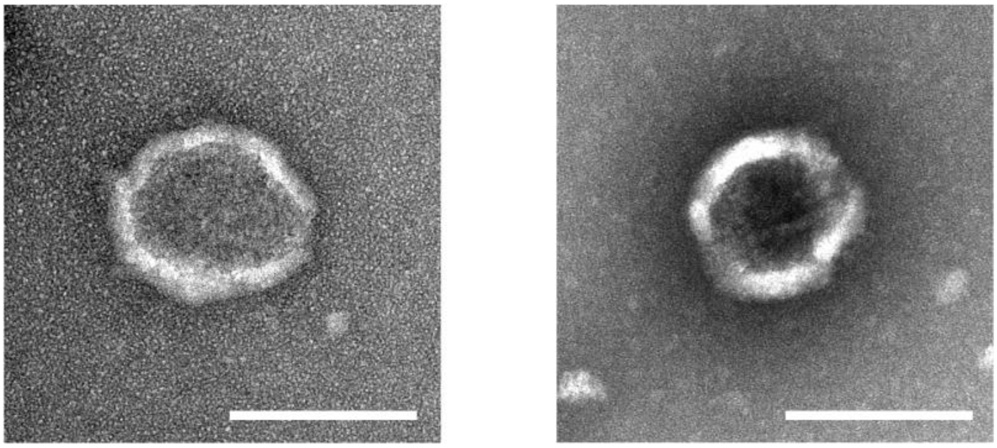

Figure S11. Transmission electron microscopy (TEM) image of TNVs (left) and PNVs (right) (Scale bars: 100 nm).

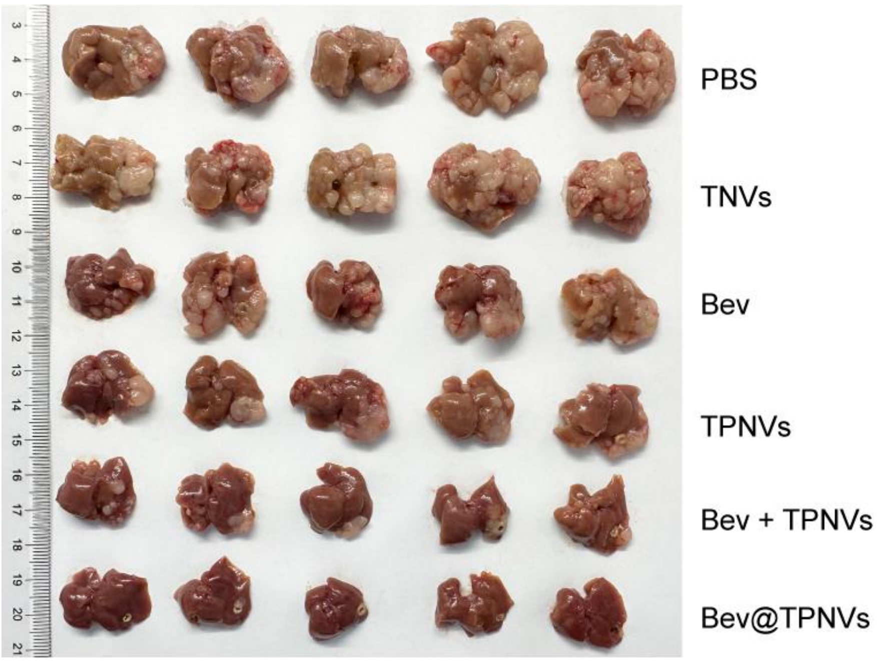

Figure S12. Representative gross images mice livers on the 18th day after different treatments.

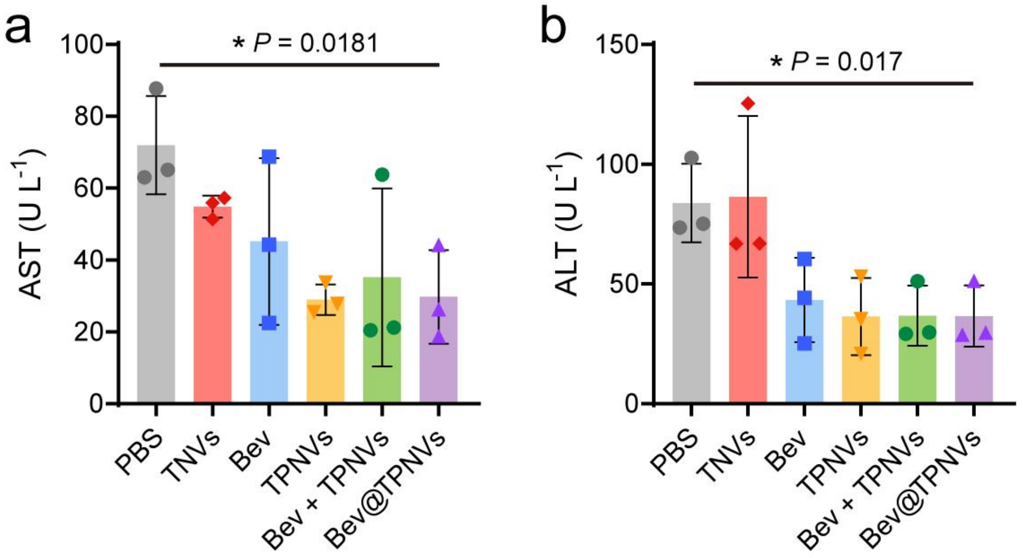

Figure S13. (a, b) Serum biochemistry data measured including aspartate aminotransferase (AST) (a) and alanine aminotransferase (ALT) (b) (n = 3). Statistical significance was calculated using two-tailed unpaired Student's *t*-test, data are expressed as means ± SD. *$P < 0.05$.

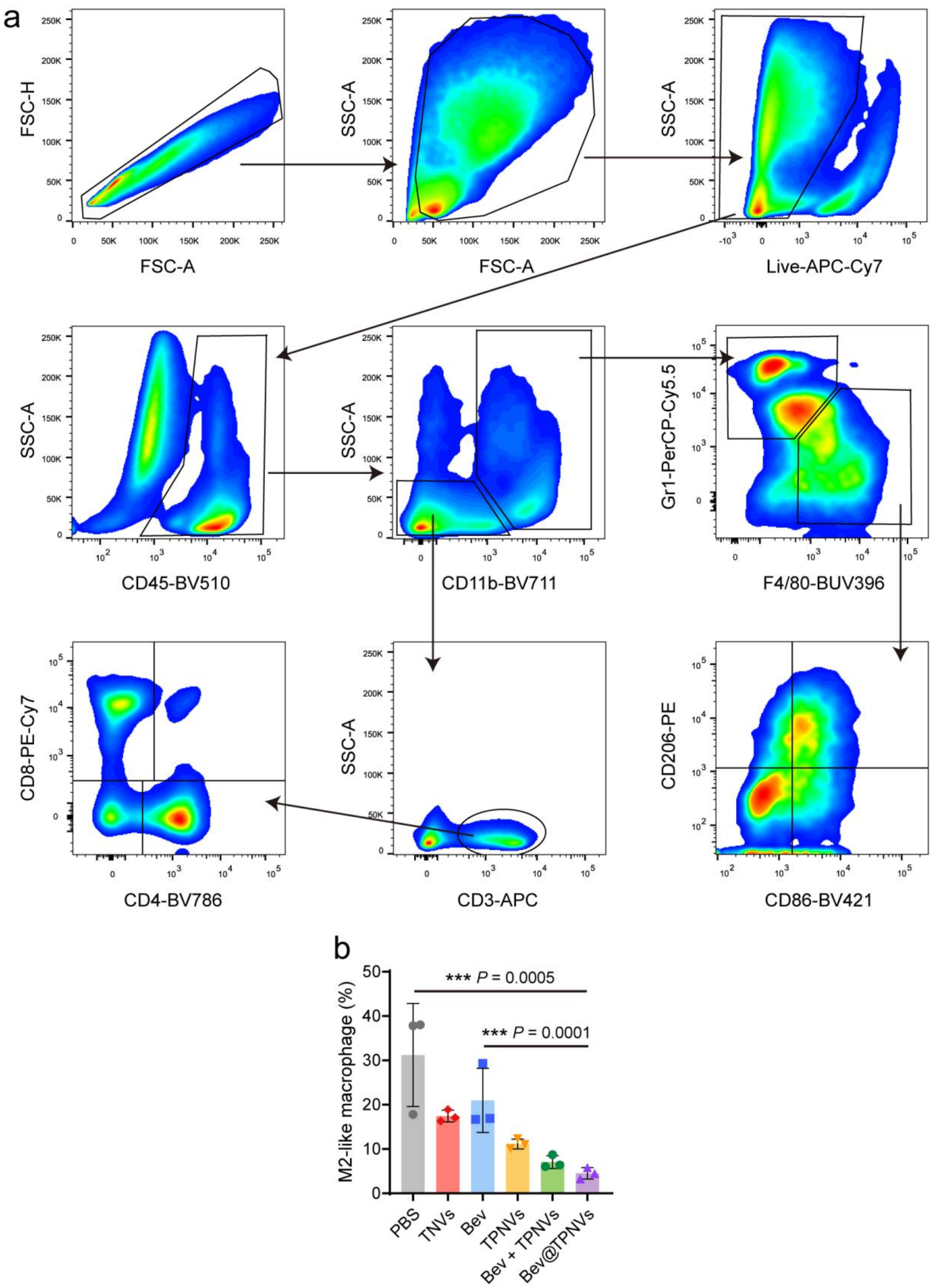

Figure S14. (a, b) Gating scheme for analyzing MDSCs, TAMs-M1, TAMs-M2 and $CD8^+$ T cells in the tissue of various treatments using flow cytometry (a) and Quantification of TAMs-M2 ($CD206^+$) in $CD11b^+F4/80^+$ cells (n = 3) (b). Statistical significance was calculated using two-tailed unpaired Student's *t*-test, data are expressed as means ± SD. ****P* < 0.001.

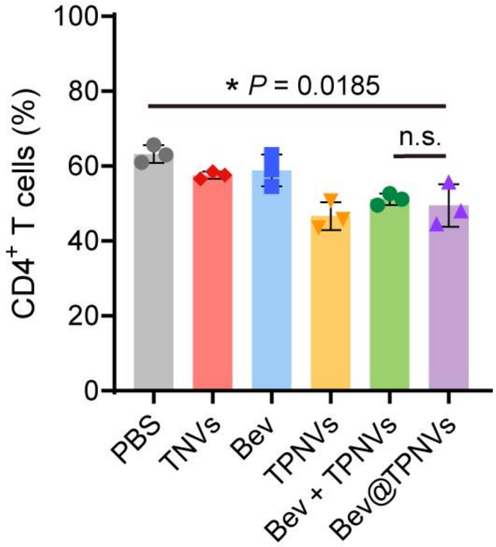

Figure S15. Quantification of $CD4^+$ T cells in $CD45^+CD3^+$ cells (n = 3). Statistical significance was calculated using two-tailed unpaired Student's *t*-test, data are expressed as means ± SD. **P* < 0.05 and n.s., not significant.

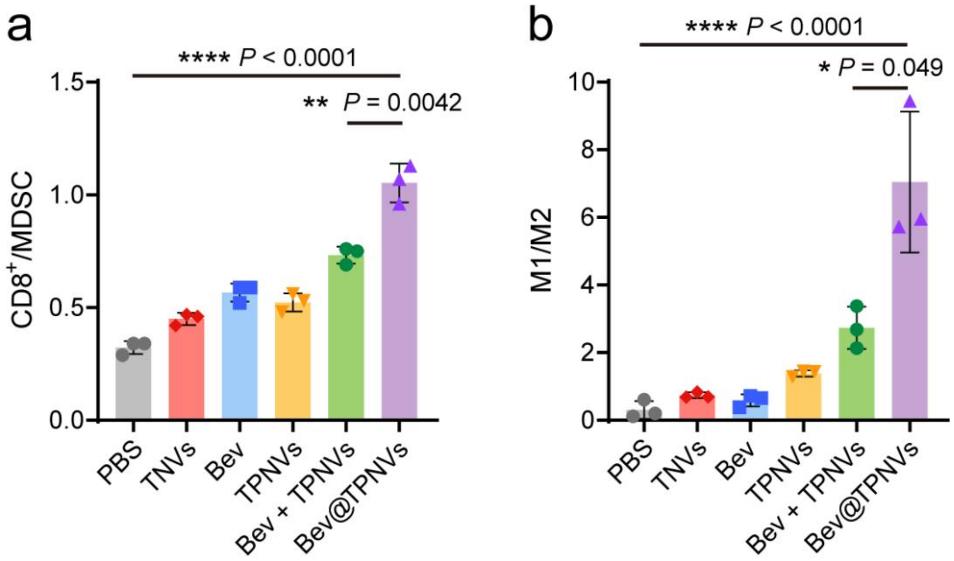

Figure S16. (a, b) Quantification by flow cytometry (FCM) of CD8^{+}/MDSC (a) and M1/M2 (b) ratios. Statistical significance was calculated using two-tailed unpaired Student's *t*-test, data are expressed as means ± SD. *$P < 0.05$, **$P < 0.01$, and ****$P < 0.0001$.

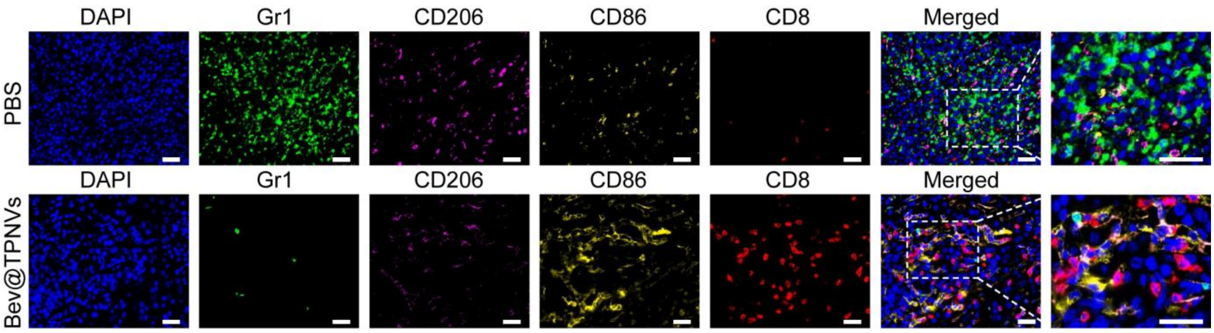

Figure S17. Representative multiplex immunohistochemistry staining images of liver sections in the reference zone (RZ) from PBS and Bev@TPNVs groups showing DAPI, Gr1^{+}, CD206^{+}, CD86^{+} and CD8^{+} cells infiltration (Scale bars: 50 μm).

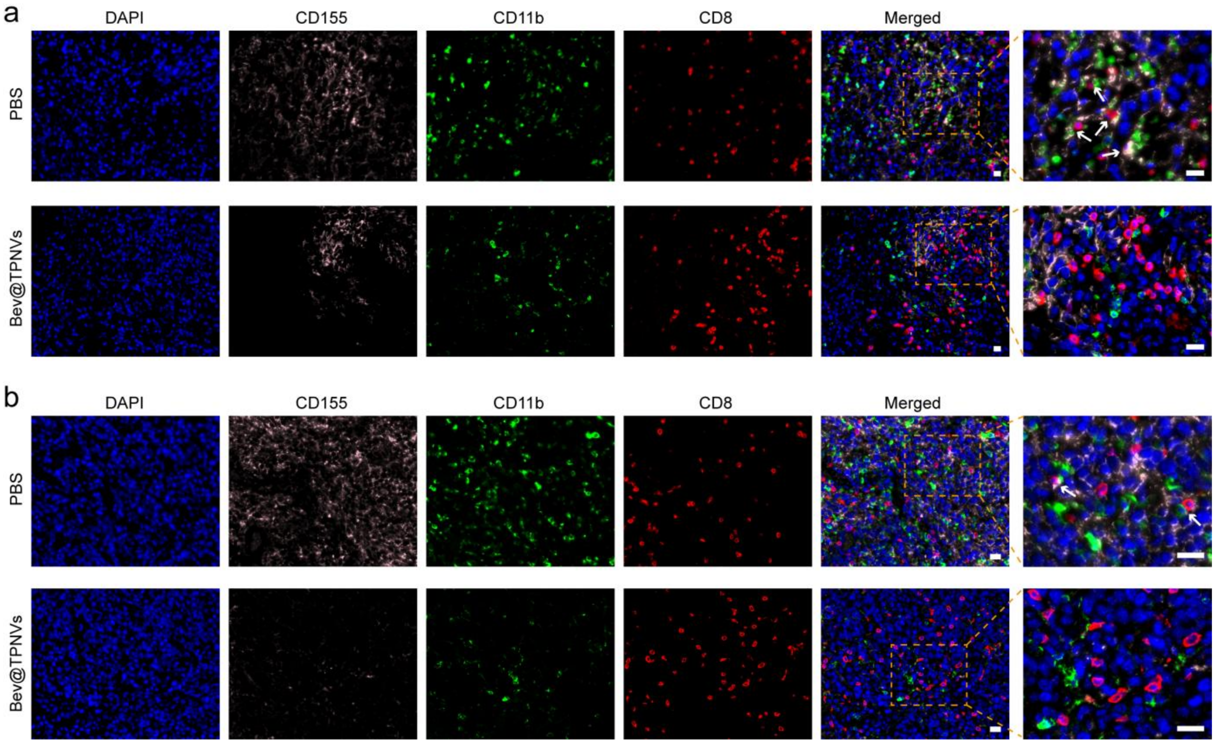

Figure S18. (a, b) Representative multiple immunohistochemistry staining images of liver sections in the transition zone (TZ) (a) and reference zone (RZ) (b) from PBS and Bev@TPNVs groups showing DAPI, CD155^{+}, CD11b^{+}, and CD8^{+} cells infiltration. The white arrows indicate the spatial co-localization of CD8^{+} T cells and CD155^{+} myeloid cells (Scale bars: 20 μm).

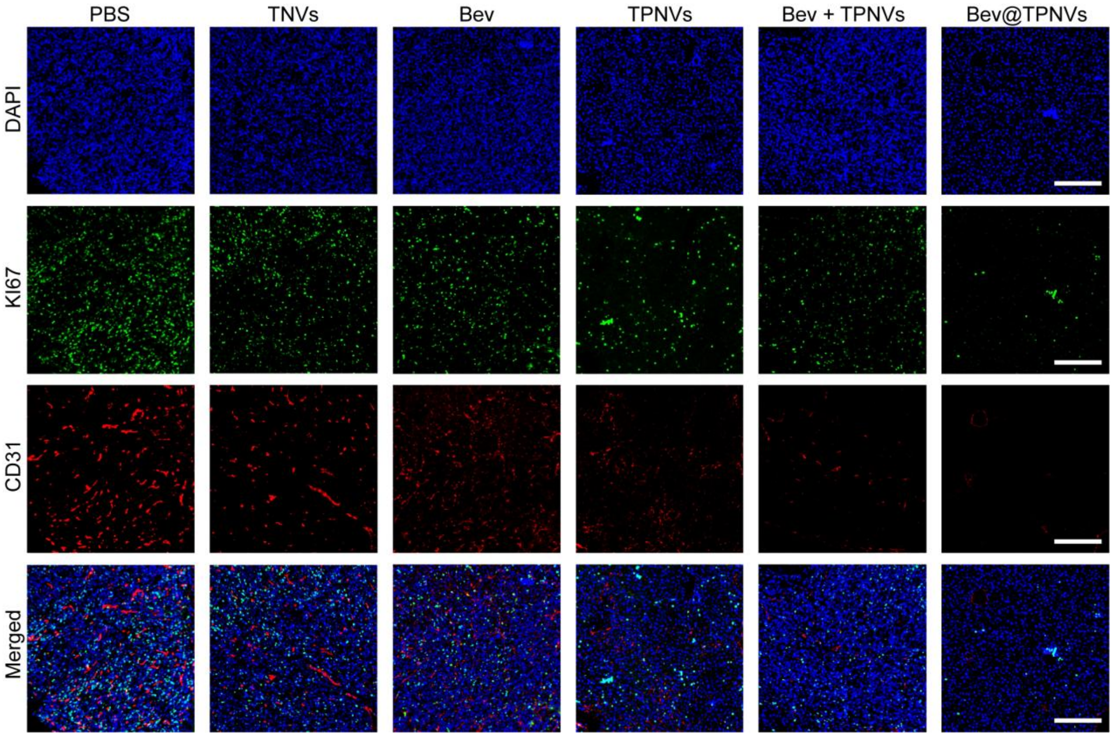

Figure S19. Representative immunofluorescence images displaying Ki67 and CD31 infiltrated liver after different treatments (Scale bars: 200 μm).

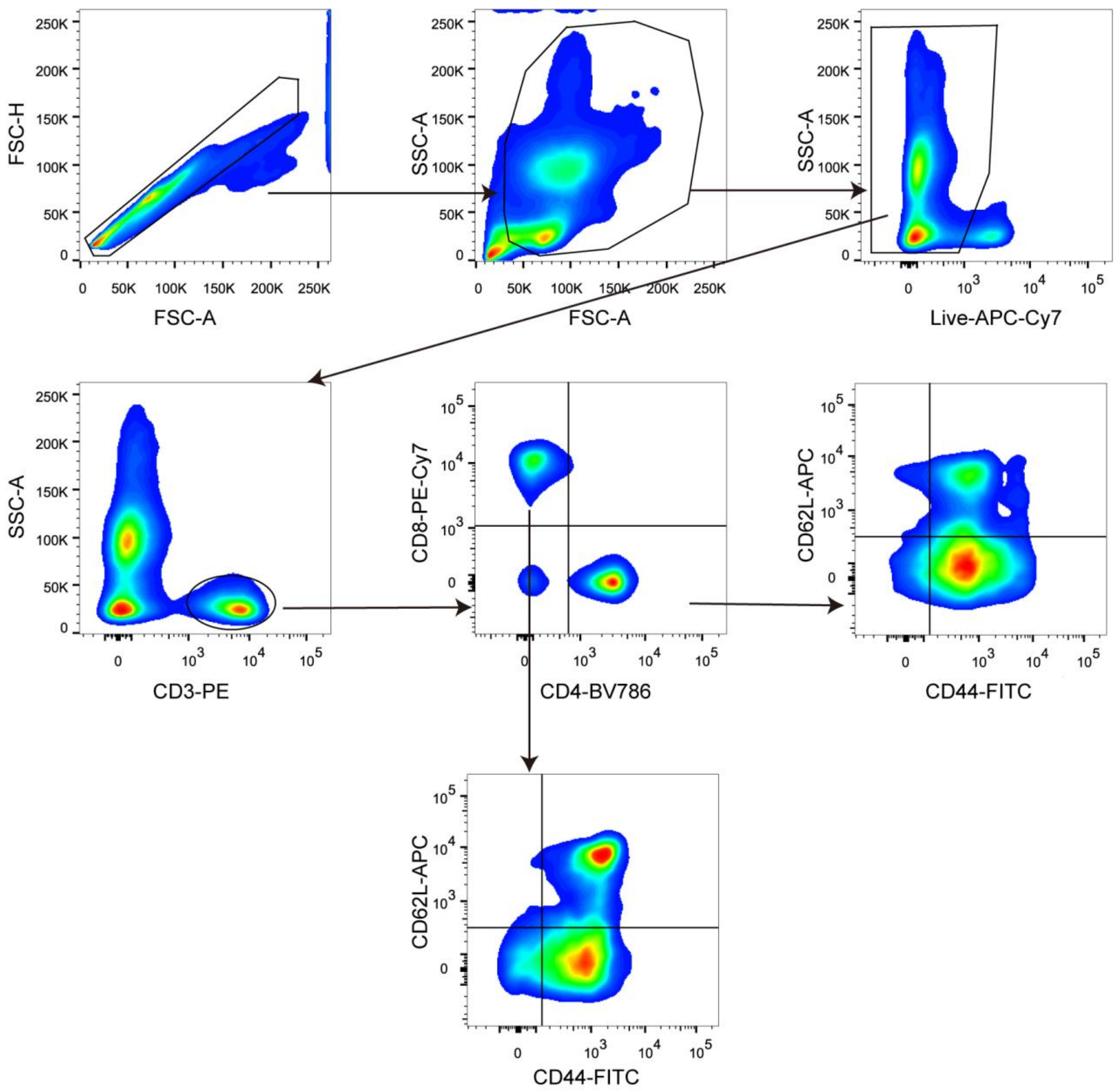

Figure S20. Gating scheme for analyzing effector memory T (Tem) cells and central memory T (Tcm) cells in the liver using flow cytometry.

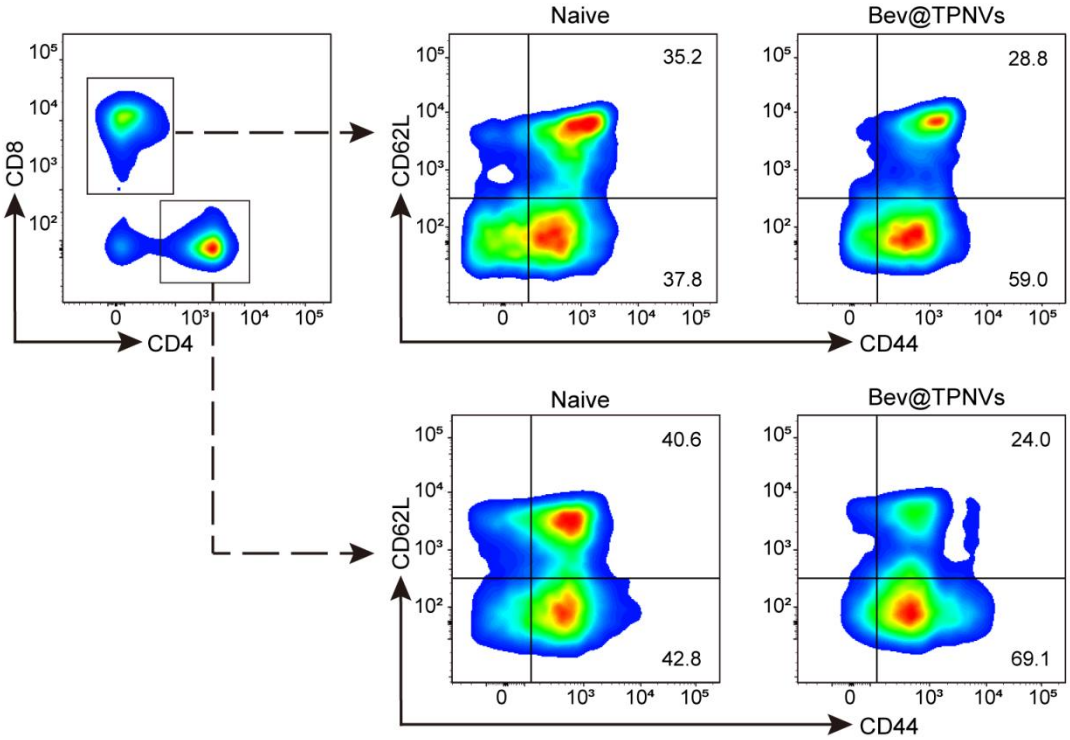

Figure S21. Representative flow cytometry (FCM) analysis of effector memory T (Tem) cells and central memory T (Tcm) cells gating on $CD45^{+}CD3^{+}$ cells in the spleens.

sample ID	new celltype	PVR anno	frequency (%)	mean UMI
Control	B cells	PVR+	5.66645	1.31818
Control	Basophils	PVR+	1.31579	1.00000
Control	Cancer cells	PVR+	55.68369	3.66568
Control	Endothelial cells	PVR+	9.11854	1.43333
Control	Myeloid cells	PVR+	25.58309	1.74929
Control	Neutrophils	PVR+	4.19753	1.17647
Control	pDC	PVR+	25.05219	1.65000
Control	Plasma cells	PVR+	5.40541	2.66667
Control	T&NK cells	PVR+	17.80938	1.50380
MWA	B cells	PVR+	5.22851	1.37037
MWA	Basophils	PVR+	3.44828	1.33333
MWA	Cancer cells	PVR+	70.05988	6.11966
MWA	Endothelial cells	PVR+	25.45455	1.73214
MWA	Myeloid cells	PVR+	50.00000	2.43169
MWA	Neutrophils	PVR+	1.46843	1.00000
MWA	pDC	PVR+	54.96829	2.18462
MWA	Plasma cells	PVR+	3.12500	1.50000
MWA	T&NK cells	PVR+	21.86782	1.64177

Table S1. Cell frequency and mean unique molecular identifier (UMI) counts.
